# Triple-feature fusion from UAV multispectral imagery enhances species-level mangrove carbon assessment

**DOI:** 10.1038/s41598-026-40303-1

**Published:** 2026-03-01

**Authors:** Yu Chen, Xiaoxue Shen, Chunhua Yan, Biqian Jiang, Ruili Li, Minwei Chai

**Affiliations:** 1 School of Environment and Energy, Shenzhen Graduate School, Peking University, Shenzhen, 518055 China; 2 Guangdong Mangrove Engineering Technology Research Center, Peking University, Shenzhen, 518055 China; 3 School of Ecology, Sun Yat-sen University, Guangzhou, 510275 China; 4 Shenzhen Campus of Sun Yat-sen University, Shenzhen, 518107 China

**Keywords:** Carbon stock, Mangrove, Species level, UAV multispectral technology, Environmental monitoring, Wetlands ecology, Forest ecology

## Abstract

Accurate estimation of mangrove ecosystem carbon stocks is essential for effective blue carbon management. Significant interspecific variations in carbon storage capacity and estimation methods arise due to species-specific biophysical characteristics, highlighting the need for precise mangrove species identification and species-level carbon stock assessment. However, limited studies assessed mangrove carbon stocks at species-level. This study, conducted in the Gaoqiao Mangrove Nature Reserve in Zhanjiang, Guangdong Province, applied UAV multispectral technology to simultaneously acquire spectral, structural and textural vegetation feature variables for mangrove species identification and established species-specific carbon stock models, thereby achieving species-level carbon stock estimation. Results showed that (1) by integrating spectral and structural features, the study achieved 89.87% overall accuracy in species identification. (2) Species-level carbon stock estimation models, incorporating spectral, structural and textural feature variables alongside field-measured carbon data, demonstrated strong predictive performance (R^2^ = 0.48-0.95). (3) The most effective vegetation feature variables for carbon estimation varied significantly across species, emphasizing the necessity of accounting for species heterogeneity in mangrove carbon stock estimations. (4) Carbon stocks exhibited significant interspecific variation, with *Rhizophora stylosa* demonstrating the highest aboveground (97.06 t hm⁻^2^) and belowground (37.22 t hm⁻^2^) stocks, compared to *Aegiceras corniculatum’*s minimum values of 49.14 and 19.88 t hm⁻^2^, respectively. This study established a UAV-based multispectral framework for mangrove species-level carbon stock estimation and provided new insights for mangrove carbon assessment and management by demonstrating the importance of considering species-specific influences on carbon stocks and their estimation.

**Supplementary Information:**

The online version contains supplementary material available at 10.1038/s41598-026-40303-1.

## Introduction

Blue carbon, defined as the sequestration of carbon via marine processes and coastal ecosystems, captures a significant portion of human-derived carbon emissions^1^. Mangroves are among the most carbon-rich and productive blue carbon ecosystems on Earth, storing about 1023 t C/ha and contributing 10–15% of global sediment carbon stocks^2,3^. Therefore, accurate estimation of carbon storage in mangrove ecosystems is crucial for blue carbon management and the development of strategies to reduce emissions.

Mangrove carbon stocks are significantly influenced by species diversity^4,5^, with significant interspecific differences in carbon storage capacity and estimation approaches, emphasizing the importance of identifying mangrove species to accurately estimate carbon stocks for different species^6,7^. However, most current studies estimate mangrove carbon stocks at the ecosystem level, with relatively scarce studies focus on the estimation of carbon stocks for individual mangrove species. The commonly-used methods for mangrove carbon stock observations include ground surveys, satellite remote sensing, and UAV technology. Ground surveys are labor-intensive, time-consuming and costly^8^. Satellite remote sensing provides long-term, dynamic, and continuous observation for large-scale mangrove monitoring^9^, so it has been widely employed to assess above-ground carbon (AGC) across many regions worldwide^10^. However, coarse-resolution imagery often lacks the resolution and accuracy required for smaller mangrove areas and species identification^11^. High-resolution and very-high-resolution data enhance resolution, but the high-resolution data lack shortwave infrared (SWIR), a wavelength critical for estimating forest biomass^12^, and is often more costly and requires advanced processing techniques^13^.

UAVs can be equipped with various sensors to capture a broader range of spectral bands than satellite sensors for mangrove species identification and carbon stock estimation, and can operate below cloud cover, thereby effectively mitigating cloud interference as well as the resolution limitations of satellite remote sensing. However, the vegetation feature variables used for carbon stock estimation are usually confined to specific aspects, making them relatively one-dimensional^14,15^. UAV visible light sensors have been applied to estimate the above-ground biomass of some small mangrove area^16,17^, but they capture only three bands: red (R), green (G), and blue (B)^18^, providing restricted spectral information and limited structural data. Hyperspectral sensors capture detailed spectral data ranging from visible light to near-infrared (NIR) or SWIR, demonstrating significant effectiveness in mangrove species identification^19,20^ and forest biomass estimation^21–23^. However, hyperspectral images typically have lower spatial resolution than visible light images, and the excess spectral data can lead to overfitting in biomass inversion models^24^ . UAV LiDAR can capture three-dimensional ground data^25^, and is used for mangrove biomass estimation as a standalone data source^26–28^ or combined with other data, such as WorldView-2^14^, ALOS-2 PALSAR-2^29^, Landsat 8^30^, GF-2 GF-3^31^, and Sentinel-1 Sentinel-2^32^. However, LiDAR offers only structural data while lacking spectral information, and the complex management and processing of LiDAR data restrict its widespread use^33^.

Therefore, there is a lack of research on simultaneously capturing and integration of spectral, textural, and structural features to accurately estimate carbon storage at the species level across different mangrove species combinations. UAV multispectral technology can effectively capture these three features. Multispectral sensors capture data in R, G, B, red-edge (RE), and NIR bands, providing more detailed spectral information compared to visible light sensors through internal modifications or lens filters^34^. UAV multispectral technology also shares advantages with UAV LiDAR, such as generating 3D point clouds, DSMs, and orthophotos from high-resolution imagery. It has been successfully applied to mangrove species identification^35–37^ and forest biomass estimation^38–40^. Despite these advances, UAV multispectral technology’s application in mangrove carbon stock estimation remains limited.

This study aimed to develop an improved method for species-level carbon stock estimation of mixed mangrove communities with UAV multispectral technology. The specific objectives include: (1) identify mangrove species based on the spectral bands and vegetation feature variables; (2) establish species-specific models for carbon stock estimation; (3) estimate the carbon stocks of mangroves at species level and explore their spatial distribution patterns. The results provided a new perspective for assessing the carbon sequestration potential of mangroves and theoretical support for the conservation and restoration of mangroves and blue carbon management.

## Materials and methods

### Study area

**Fig 1:**
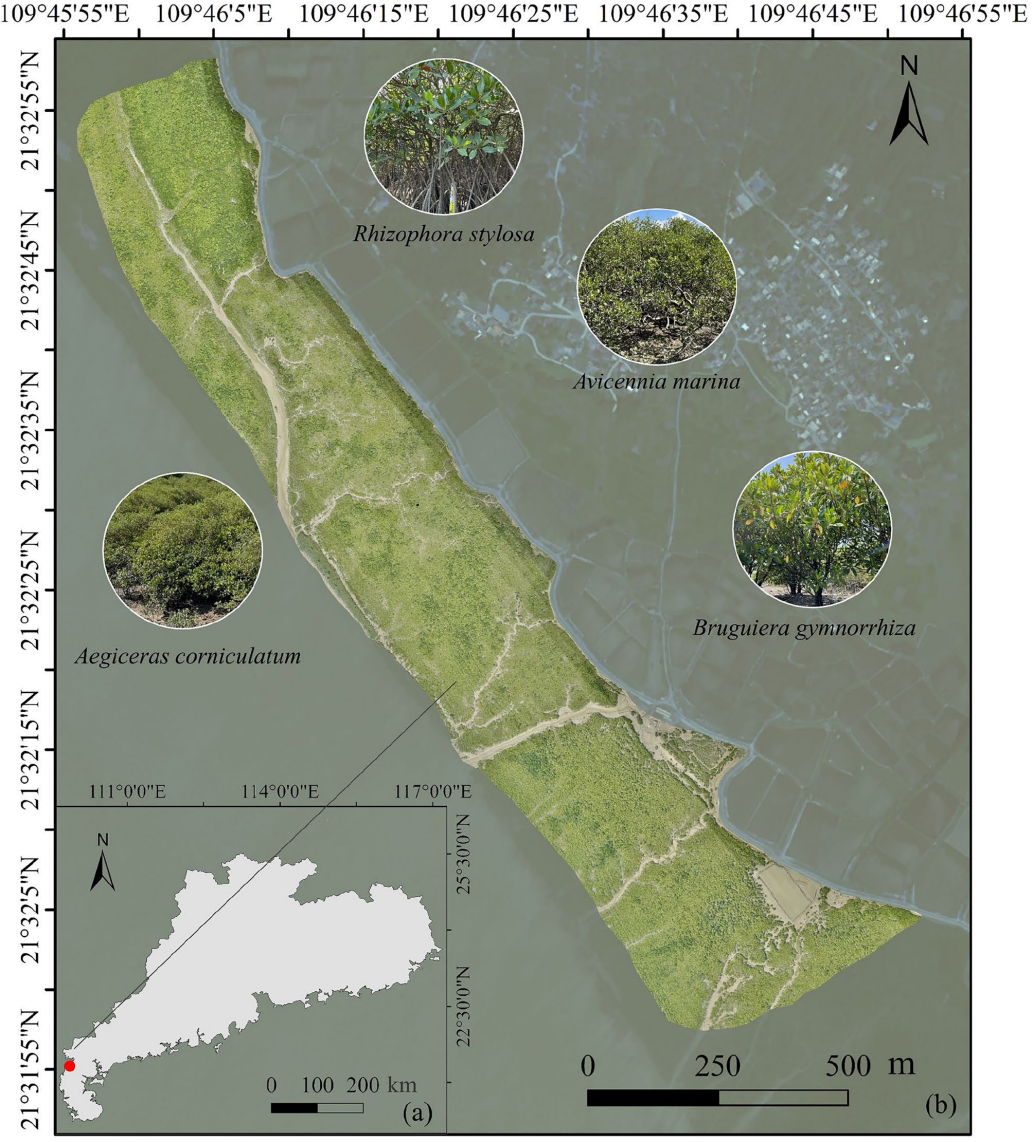
Overview of the study area. (**a**) The location of the study area in Guangdong Province, China; (**b**) UAV orthomosaic imagery of the study area and the dominant mangrove species. The main dominant species includes *R. stylosa, A. marina, A. corniculatum* and *B. gymnorrhiza.* The map was created by the authors using ArcGIS Desktop 10.8.1 (Esri, Redlands, CA, USA; https://www.esri.com/software/arcgis) based on Sentinel-2 imagery provided by the Copernicus Programme of the European Space Agency (ESA). The UAV imagery was acquired and processed by the authors.

The study area is located in the Gaoqiao Mangrove Nature Reserve in Zhanjiang, Guangdong Province, the largest mangrove nature reserve in China (Fig. 1). The region has a tropical monsoon climate, with an average annual temperature of 22.4°C and an annual rainfall of approximately 1816 mm. The rainy season occurs between April and September, and the average annual seawater temperature is 23.5°C. The area experiences a diurnal tidal regime, with an average tidal range of 2.53 m and a maximum tidal range of approximately 6.25 m^41^. The study site (109°45’-109°47’E, 22°31’-22°33’N) covers approximately 71 hectares within the reserve. The Gaoqiao mangrove ecosystem has a complex community structure, consisting of both natural and artificial mangrove areas. The dominant mangrove species include *Rhizophora stylosa*, *Avicennia marina*, *Aegiceras corniculatum* and *Bruguiera gymnorrhiza* (Fig. 1). The main community types include *R. stylosa*, *R. stylosa*-* A. marina*, *A. marina*, *A. marina*-*A. corniculatum*, *A. marina*-*B. gymnorrhiza*, *A. corniculatum*, *B. gymnorrhiza*, and *B. gymnorrhiza*-*R. stylosa* communities. Mangroves in the Yingluo Bay are distributed in a coastal strip, with the main species distributed as follows: *B. gymnorrhiza* primarily in high tidal zones, *R. stylosa* in high and mid-tidal zones, *A. corniculatum* and *A. marina* across high, mid, and low tidal zones. In this study, tidal zones were determined based on tidal cycles and tidal heights and were approximately classified as high (>3 m), mid (1–3 m), and low (<1 m) intertidal elevations.

### Ground survey

Ground data were collected from November 5 to 7, 2021, across 40 sample plots (5 m × 5 m), comprising four mangrove species with 10 plots allocated to each species. Permission to conduct the field survey was obtained from the Gaoqiao Mangrove Nature Reserve in Zhanjiang, Guangdong Province. The plots were randomly established within each species and were distributed across high-, mid-, and low-tidal zones. The geographic location of each plot was recorded using a DJI D-RTK 2 Mobile Station, a high-precision GNSS receiver providing centimeter-level positioning accuracy. Ground data included species composition, tree count, geographic location, and individual tree attributes such as height, DBH or basal diameter, and canopy diameter. DBH was measured at 1.3 m above the ground. For multi-stemmed mangroves, if the bifurcation occurred above this height, DBH was measured at the bifurcation point; otherwise, the diameter of each stem was measured separately and treated as individual trees. Basal diameter was measured at 30 cm above the ground. Mangrove above-ground biomass (AGB) and below-ground biomass (BGB) were estimated using species-specific allometric growth equations. Carbon conversion factors were applied to derive above-ground carbon stock (AGC) and below-ground carbon stock (BGC) (Table S1 in the Supplementary file)^42^. Table 1 summarizes the ground survey results, detailing the mangrove community structures within the study area.

**Table 1:** Ground survey results of different mangrove community structures.

Species	n	Height (m)	Canopy (m)	DBH (cm)	Density (trees·hm-2)		AGC (t·hm-2)	BGC (t·hm-2)
*A. marina*	50	2.44 ± 0.50	2.87 ± 0.91	7.45 ± 2.82	9280 ± 2152	33.54 ± 8.59		23.26 ± 6.84
*A. corniculatum*	54	1.83 ± 0.46	1.39 ± 0.43	-	133200 ± 48745	40.24 ± 15.16		16.22 ± 6.11
*R. stylosa*	46	4.73 ± 0.72	3.18 ± 1.34	13.85 ± 10.05	7480 ± 3514	96.27 ± 50.50		33.55 ± 17.93
*B. gymnorrhiza*	50	2.51 ± 0.65	2.19 ± 0.62	13.51 ± 5.95	8320 ± 3835	69.26 ± 41.10		24.62 ± 12.18

### UAV survey

Multispectral imagery was collected from November 6 to 9, 2021. Before UAV flights, ground control points (GCPs) were placed throughout the study area for geo-referencing purposes. The ground markers were evenly distributed across the study area, and the central points of the markers were recorded using a DJI D-RTK 2 high-precision station in fixed solution mode. To ensure accuracy, UAV flights were conducted under clear, low-wind conditions with minimal cloud cover, and during low tide. A DJI P4 Multispectral UAV was used, which was equipped with a multispectral sensor capable of capturing data in B, G, R, RE, and NIR bands (detailed in Table S2). Flight missions were planned using the DJI GS Pro software (iOS v. 2.0.13), at an altitude of 80 m, with an 80% forward overlap and a 75% side overlap. Data were acquired every 2 seconds under RTK fixed solution mode, and flight parameters remained consistent throughout.

A total of 7868 multispectral images were pre-processed using DJI Terra (v.3.1.4). The workflow included data import, triangulation, and multispectral 2D stitching. The final output comprised a digital orthophoto model (DOM), digital surface model (DSM), and various vegetation indices, including normalized difference vegetation index (NDVI), green normalized difference vegetation index (GNDVI), normalized difference red-edge vegetation index (NDRE), optimized soil-adjusted vegetation index (OSAVI), leaf chlorophyll index (LCI), along with the individual spectral bands. The spatial resolution of the UAV-based multispectral images was 0.045 m, which resulted in extremely large data volumes. To simplify computation, a final resolution of 0.53 m was selected as the most suitable for meeting the requirements of this study, as resampling to approximately 0.5 m allows each pixel to integrate key vegetation features while remaining smaller than the average mangrove canopy diameter.

### Calculation of vegetation feature variables

Spectral, structural, and textural features were extracted from the UAV-based multispectral images for mangrove species identification and carbon stock estimation.

#### Spectral features

 Spectral features were categorized into raw spectral values and vegetation indices derived from UAV multispectral imagery. Vegetation indices combine pixel values from two or more spectral bands using various algorithms, primarily focusing on band ratios or feature scaling. In addition to the commonly used R, G, and B bands, we incorporated NIR and RE bands, which are closely associated with species identification and biomass estimation^43^. The vegetation indices used in this study include NDVI, GNDVI, NDRE, OSAVI, and LCI, which were derived from pre-processed UAV multispectral data, as well as the ratio vegetation index (RVI), modified anthocyanin content index (MACI), red-edge simple ratio index (VREI), and simple ratio (SR) vegetation index (Table S3). NDVI and GNDVI characterize vegetation vigor and photosynthetic activity, with GNDVI showing enhanced sensitivity to chlorophyll content; NDRE and VREI exploit RE information and are suitable for high-biomass or high-chlorophyll vegetation by reducing saturation effects; OSAVI minimizes soil background influence and performs well under sparse vegetation cover; LCI, RVI, and SR are closely related to chlorophyll concentration and biomass, making them effective indicators for carbon stock estimation; and MACI reflects plant stress conditions that may indirectly affect carbon sequestration potential^43^.

#### Structural features

 Structural features focused on the vertical characteristics of vegetation, particularly canopy height (H_Mean). Canopy height was derived using a canopy height model (CHM), calculated as the difference between the vegetation surface elevation (DSM) and the ground elevation (DTM). While the DSM is automatically generated during UAV modeling, the DTM was manually created using inverse distance weighting (IDW) interpolation. The accuracy of the DTM was assessed using ground-measured GCPs and evaluated via root mean square error (RMSE) (Table S4). The lowest RMSE was achieved when the pixel size was 3.5, the power was 3, and the number of points was 3. Using the most accurate DTM, the CHM was computed. A comparison between UAV-derived tree heights and ground-measured heights showed a strong correlation (R^2^ = 0.95, RMSE = 0.49 m).

#### Textural features

 Textural features describe the spatial arrangement of pixel intensity variations within an image, capturing characteristics such as smoothness, uniformity, and variability^44^. Different mangrove species exhibit distinct textural patterns due to differences in leaf arrangement, branch density, height, and size. Textural analysis enhances both species identification accuracy^45^ and biomass estimation^46^. In this study, textural features were extracted using the gray-level co-occurrence matrix (GLCM) method^44^. Eight textural metrics were calculated for each of the five spectral bands using ENVI (v.5.3): Mean (Mean), Variance (Var), Entropy (Ent), Dissimilarity (Dis), Homogeneity (Hom), Contrast (Con), Correlation (Cor), and Angular Second Moment (Asm) (Table S5).

### Mangrove species identification

The use of 2D information, including raw spectral values and vegetation indices, allows for relatively accurate classification of mangrove species^47^. Meanwhile, incorporating 3D structural features further enhances classification accuracy^48^. To provide accurate distribution information of mangrove species for the construction of carbon stock estimation models, 15 combinations of spectral and structural feature variables (V1–V15, Table S6) were used as input for mangrove species identification. A pixel-based classification method was employed to identify mangrove species, with training and validation samples collected from multispectral images through visual interpretation. A total of 1,320,641 labeled pixels were obtained, of which 70% were used for model training and 30% for validation. The maximum likelihood classification was used for species classification, since it was proved well-performed in pixel-based classification method and has been applied to mangrove species classification^49,50^. The results were evaluated using a confusion matrix. Key assessment metrics included overall accuracy (OA), producer’s accuracy (PA) and user’s accuracy (UA) for each land cover type. PA reflects the model’s ability to correctly identify reference samples of each mangrove species, while UA indicates the reliability of the classified map by quantifying commission errors. If classification accuracy was unsatisfactory, training samples were refined, and the process was iteratively repeated until optimal results were achieved. The pixel-based classification analysis was conducted using ENVI (v.5.3) software.

### Development of species-level carbon stock models

#### Model development for mangrove carbon stock estimation

**Fig 2:**
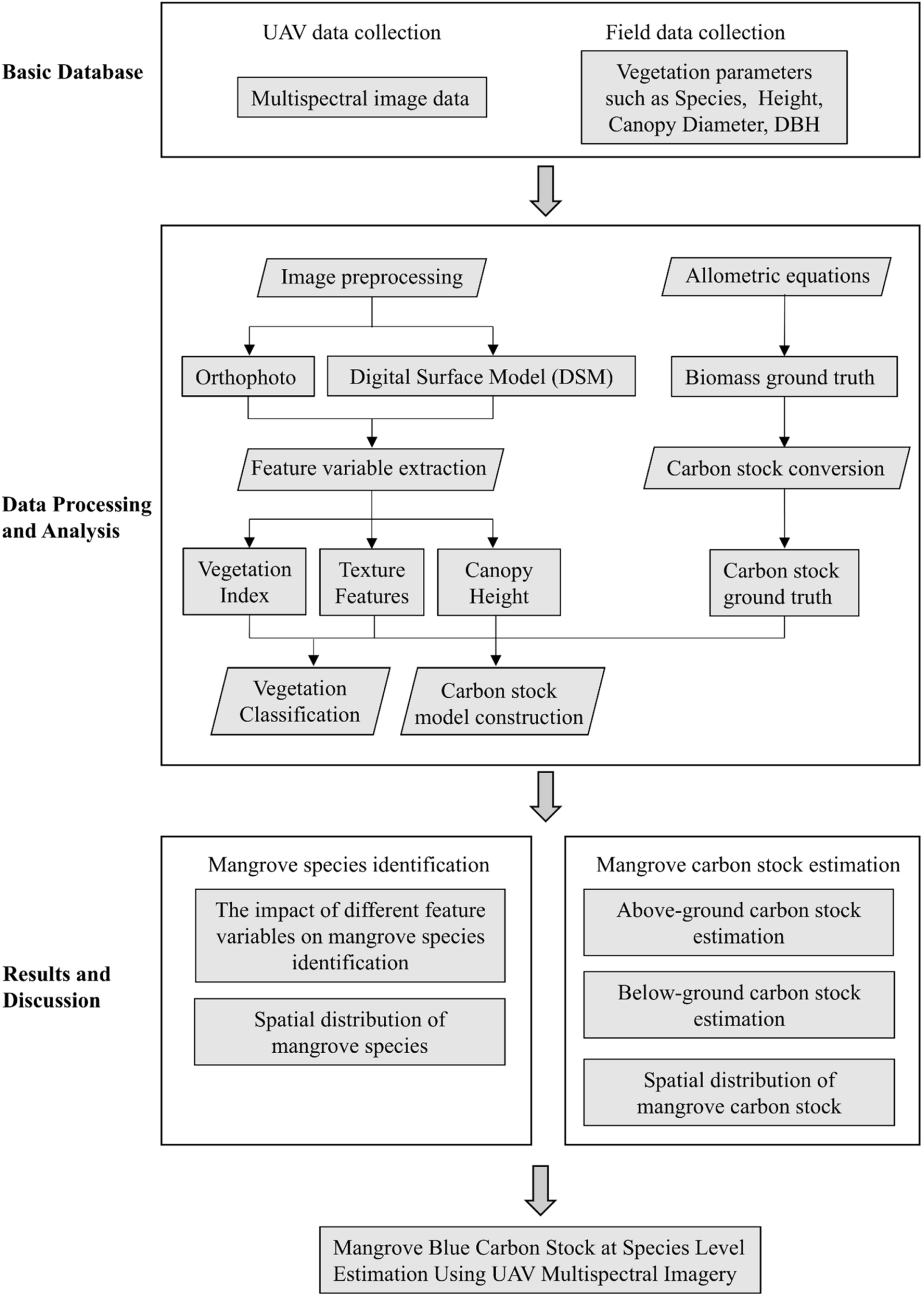
The work process of estimating species level mangrove carbon stock through the integration of UAV multispectral data and field analysis.

Spectral, structural and textural features have been proved effective for carbon stock estimation^17,51^. This study investigated the statistical relationships between these features (Table 2) and carbon stocks across different mangrove species and species combinations (Fig. 2). The UAV-based data at 0.53 m resolution were aggregated by averaging all pixels within each plot to match the 5 m resolution of the ground survey sample plots. Firstly, Pearson correlation was explored between vegetation feature variables and carbon stocks. Secondly, the variable with the highest correlation to mangrove carbon stocks was selected to construct univariate linear regression models for estimating above-ground carbon (AGC) and below-ground carbon (BGC) for each mangrove species and species combinations. Specifically, for species-combination models, ground-survey plots and corresponding UAV-derived variables from two species (e.g., *R. stylosa* and *A. marina*) were pooled and jointly used as inputs to train a species-combination model. Thirdly, multivariate linear regression models were constructed to improve the performance of those underperforming models. In the multivariate model, variance inflation factors (VIF) were used to detect and reduce multicollinearity among independent variables. A VIF threshold of 10 was applied to identify multicollinearity, and only models with VIF < 3 were selected to ensure robust parameter estimation. Additionally, only models with statistically significant relationships (P < 0.05) were retained. We realized that machine learning has been a prevailing tool for this type of application. However, the small sample sizes (only 10 plots for each species) owing to intensive labor and limited study area constrained our use of tree-based or nonparametric methods due to the risk of overfitting, and the interpretability of linear models could be relatively straightforward^52,53^. All analyses were conducted using SPSS (v.18.0).

#### Model validation

**Table 2:** The predictor variables of mangrove carbon stock models based on UAV.

Variable type	Variables							
Spectral features	NDVI	NDRE	GNDVI	OSAVI	LCI	MACI	RVI	VERI
	SR							
Structural feature	H_Mean							
Texture features	R_Con	R_Cor	R_Dis	R_Ent	R_Hom	R_Mean	R_Asm	R_Var
	G_Con	G_Cor	G_Dis	G_Ent	G_Hom	G_Mean	G_Asm	G_Var
	B_Con	B_Cor	B_Dis	B_Ent	B_Hom	B_Mean	B_Asm	B_Var
	RE_Con	RE_Cor	RE_Dis	RE_Ent	RE_Hom	RE_Mean	RE_Asm	RE_Var
	NIR_Con	NIR_Cor	NIR_Dis	NIR_Ent	NIR_Hom	NIR_Mean	NIR_Asm	NIR_Var

Leave-one-out cross-validation (LOOCV) was employed to assess model performance. In LOOCV, each data point is sequentially used as the test sample while the remaining data serve as the training set, ensuring every point is tested exactly once^54^. Model fit and predictive accuracy were evaluated using the coefficient of determination (R^2^) and the root mean square error (RMSE) as evaluation metrics. Higher R^2^ and lower RMSE values indicate better model performance, predictive accuracy, and robustness.

#### Carbon stock estimation of the study area

 Among all the carbon stock estimation models constructed, the models that were able to encompass all mangrove species in the study area and demonstrated superior performance was selected to estimate mangrove carbon stocks of the study area and explore their spatial distribution patterns.

## Results

### Mangrove species identification

Evaluation metrics, including OA, PA and UA for each land cover (Table 3), were calculated to assess species classification accuracy of across 15 combinations of spectral and structural features. As shown in Table 3, the inclusion of RE and NIR bands from UAV multispectral imagery significantly improved species identification accuracy. Compared to the commonly-used combination of R, G, and B bands (V1), adding the RE band (V2) increased the PA for *B. gymnorrhiza* from 25.45% to 32.43%, while the NIR band (V3) enhanced the distinction of *A. marina* raising its PA from 77.01% to 82.28%. When both RE and NIR bands were included (V4), the PA for *B. gymnorrhiza* and *A. marina* further improved to 52.22% and 87.23%, respectively, reducing patch fragmentation and enhancing soil and water differentiation. This resulted in an OA increase from 81.25% to 85.12%.

**Table 3:** Overall accuracy (OA), producer’s accuracy (PA) and user’s accuracy (UA) calculated from the identification results.

Combination	OA (%)	Land cover PA and UA (%)													
		Soil		Water		*R. stylosa*		*B. gymnorrhiza*		*A. marina*		*A. corniculatum*		Shadow	
		PA	UA	PA	UA	PA	UA	PA	UA	PA	UA	PA	UA	PA	UA
V1	81.25	98.46	96.25	91.25	94.01	96.35	79.25	25.45	35.26	77.01	83.85	41.32	66.84	79.85	89.28
V2	82.63	96.47	96.03	90.33	87.04	94.76	84.88	32.43	34.81	81.19	82.93	42.97	65.53	87.88	90.10
V3	82.88	96.05	96.03	90.20	85.51	95.43	84.88	26.47	33.19	82.28	82.94	42.04	66.68	88.21	89.11
V4	85.12	96.25	95.60	87.66	85.75	93.71	89.35	52.22	54.07	87.23	83.21	43.84	67.63	88.71	90.41
V5	81.07	96.70	92.02	75.51	84.44	89.85	92.09	0.00	0.00	97.37	69.70	9.67	64.73	75.14	94.75
V6	77.90	88.49	94.57	84.72	62.03	94.03	83.10	43.07	34.99	75.83	77.66	21.40	42.28	85.56	94.34
V7	82.10	96.56	93.69	86.35	85.68	89.35	90.85	59.63	47.01	91.25	76.86	17.42	51.80	73.37	94.81
V8	82.52	98.68	96.30	91.70	96.03	90.44	91.78	39.65	41.25	80.29	79.71	56.68	53.33	85.46	92.27
V9	80.90	94.33	93.94	79.95	77.81	88.28	91.41	56.67	42.94	76.80	80.10	61.28	52.57	84.48	91.57
V10	83.91	96.91	92.46	77.86	86.19	88.99	95.28	7.94	59.71	95.98	72.88	44.02	81.15	75.62	95.26
V11	84.94	96.53	93.90	86.68	86.11	88.77	94.02	69.01	48.86	89.99	81.98	50.65	67.28	73.12	94.84
V12	86.46	98.15	95.26	88.18	91.84	93.29	95.93	74.10	66.45	80.49	88.29	75.99	53.56	80.93	95.19
V13	85.44	97.10	94.87	86.81	88.19	89.98	95.31	22.94	62.79	95.53	75.94	44.90	79.57	85.46	92.16
V14	84.99	96.41	96.19	92.36	86.32	91.19	93.42	55.26	53.06	87.73	80.13	50.14	65.60	83.18	94.26
V15	89.87	98.89	96.16	90.33	95.71	94.14	95.89	70.30	72.00	88.15	89.17	80.34	69.68	84.23	94.63

Normalized vegetation index combinations (V5, V6, V7) produced more continuous species distribution maps, with reduced fragmentation and better reflection of actual species distribution. Incorporating vegetation indices into raw spectral data (V12) reduced misclassification and omission errors in soil and water bodies while increasing the PA for *B. gymnorrhiza* from 52.22% to 74.1% and for *A. corniculatum* from 43.84% to 75.99%. The combination minimized the influence of external light variation, resulting in smoother classification outputs with reduced patch fragmentation.

Structural data can also help improve the species identification performance. Incorporating structural data into the visible light combination (V1 vs V8) and normalized vegetation index combinations (V6 vs V9, V5 vs V10, V7 vs V11) lead to a significant improved the identification of *A. marina*, *B. gymnorrhiza*, and *A. corniculatum*.

In our study, the combination of raw spectral imagery, vegetation indices, and structural data (V15) produced the best species identification result, with an OA of 89.87%. This significantly improved the classification of *A. marina*, *B. gymnorrhiza*, and *A. corniculatum*, which are spectrally similar but differ in height. The PA for the two most challenging species, *A. corniculatum* and *B. gymnorrhiza*, reached 70.3% and 80.34%, respectively. Given the high spectral similarity between *A. corniculatum* and *A. marina*, their minimal height difference, the severe community mixing in the study area, and the limited distribution of *B. gymnorrhiza* interspersed with *A. marina* and *A. corniculatum*, the species identification accuracy of the proposed model based on V15 were deemed acceptable for the objectives of this study.

### Spatial distribution of mangrove species

**Fig 3:**
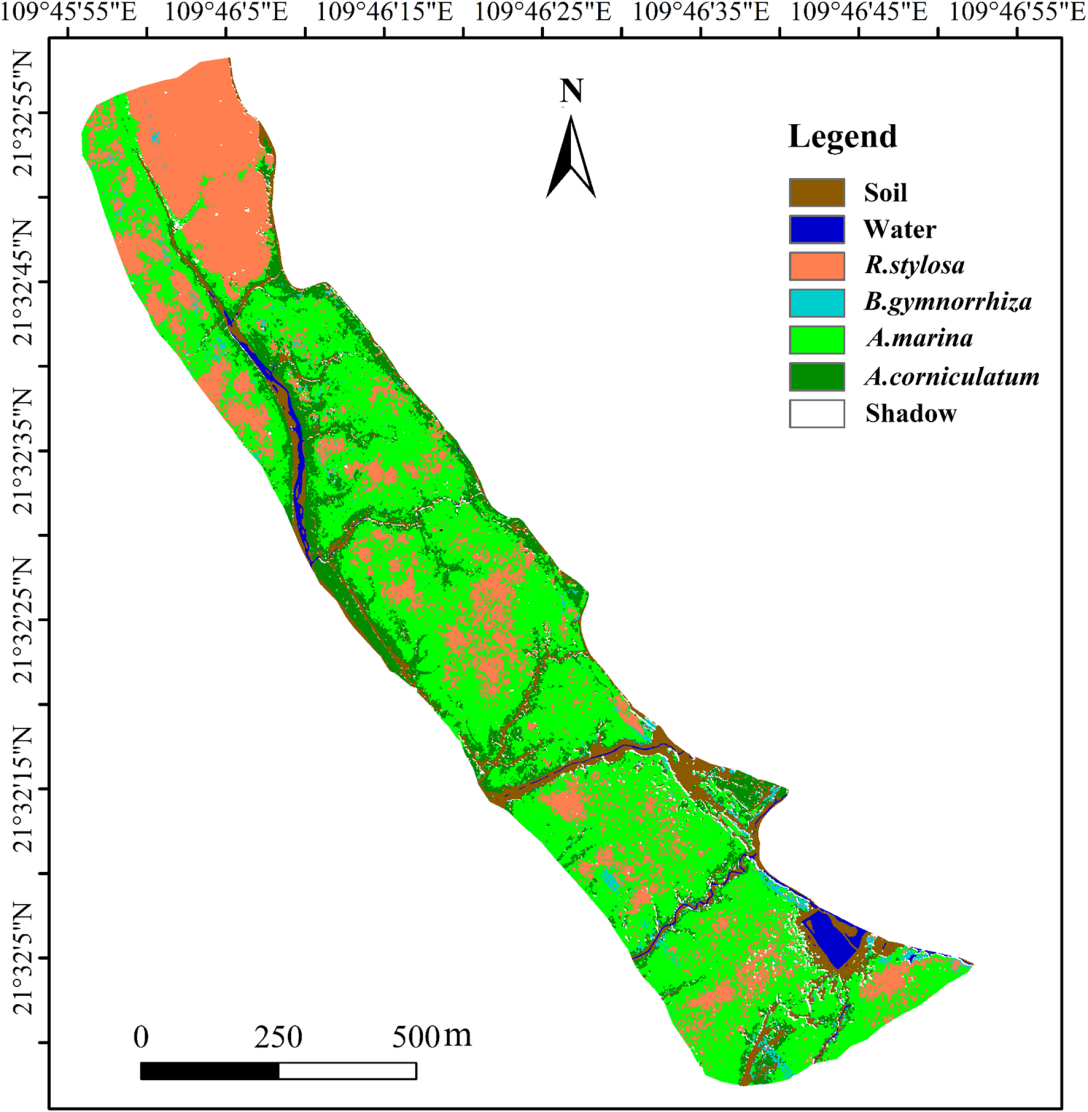
Spatial distribution of mangrove communities in the study area.

Fig. 3 showed the final distribution map of mangrove species deriving from the V15 model with the best performance. The total study area covers 70.97 hm^2^, with mangroves occupying 62.64 hm^2^. There are 4 mangrove species distribute in the area, with *A. marina* covering 35.51 hm^2^, *A. corniculatum* 8.84 hm^2^, *R. stylosa* 16.99 hm^2^, and *B. gymnorrhiza* 1.30 hm^2^. *A. marina* is widely distributed throughout the study area, from high to low tidal zones, and is the dominant species in study area, particularly in the low-tidal zone. *R. stylosa* is primarily concentrated in the northwest, forming large mature stands, with smaller patches scattered across tidal zones, often mixed with *A. marina*. *B. gymnorrhiza* is sparsely distributed, mainly in mid- to high-tidal zones, forming small clusters frequently mixed with *A. marina* and *A. corniculatum*.* A. corniculatum* is typically found along the periphery, typically forming monospecific stands along riverbanks, mudflat edges, and dikes.

### Mangrove carbon stock estimation at species level

Pearson correlation analysis has found the variable that has the strongest correlation with the AGC and BGC of 4 mangrove species (Table 4). In our study, vegetation indices such as OSAVI, VREI, and LCI, which contain R, RE, and NIR band information, showed a strong correlation with AGC and BGC of individual species. Moreover, the textural features Var, Mean, Asm, and Ent were highly correlated with AGC of mangrove species, while Var, Mean, and Ent were highly correlated with BGC (Fig. S1).

**Table 4:** The variable that has the strongest correlation with the AGC and BGC of 4 mangrove species.

Species	Carbon stock	Variable	Pearson’s r
*R. stylosa*	AGC	H_Mean	0.71*
	BGC	H_Mean	0.79**
*A. marina*	AGC	VREI	0.62
	BGC	B_Mean	0.70*
*A. corniculatum*	AGC	RE_Mean	0.84**
	BGC	RE_Mean	0.84**
*B. gymnorrhiza*	AGC	B_Var	0.92**
	BGC	B_Var	0.90**

Note: *** indicates a significant correlation at the 0.001 level (two-tailed); ** indicates a significant correlation at the 0.01 level (two-tailed); * indicates a significant correlation at the 0.05 level (two-tailed).

Based on the highest correlation feature variables, we first developed univariate linear regression models to estimate the carbon stocks of 4 mangrove species individually (Table S7). For *A. corniculatum*, the univariate model based on the textural variable RE_Mean provided a reasonable degree of accuracy (AGC: R^2^ = 0.59, RMSE = 9.71 t hm^-2^; BGC: R^2^ = 0.59, RMSE = 3.91 t hm^-2^). However, the univariate models for *R. stylosa*, *B. gymnorrhiza*, and *A. marina* showed lower estimation accuracy.

Next, the 4 mangrove species were analyzed as 11 mangrove species combinations to develop univariate models for carbon stock estimation (Table S8). For species combinations that included *A. corniculatum*, the R^2^ value of the carbon stock estimation model for *R. stylosa*-*A. corniculatum* combination was comparable to the model for individual *A. corniculatum*, while the RMSEs of *R. stylosa*-*A. corniculatum* combination model was significantly higher (AGC: R^2^ = 0.59, RMSE = 30.01 t hm^-2^; BGC: R^2^ = 0.63, RMSE = 9.76 t hm^-2^). The estimation models for other species combinations containing *A. corniculatum* exhibited relatively lower accuracy. Therefore, in this study, *A. corniculatum* was treated individually, and its carbon stock was estimated using a univariate model. Meanwhile, the univariate models of *R. stylosa*-*A. marina* and *R. stylosa*-*A. marina*-*A. corniculatum* combinations using only the H_Mean demonstrated reasonable performance. In particular, for the *R. stylosa-A. marina* combination, the regression model based on H_Mean outperformed the models developed for individual species. The fitting and validation results of this model were in close agreement, demonstrating stable performance and making it suitable for estimating carbon stock of *R. stylosa*-*A. marina* combination (AGC: R^2^ = 0.62, RMSE = 29.80 t hm^-2^; BGC: R^2^ = 0.48, RMSE = 10.48 t hm^-2^).

Multivariate models were developed to improve the performance of univariate models for other species combinations (Table S9 and S10). When the carbon stock of *B. gymnorrhiza* was estimated separately using multiple textural variables, the model exhibited strong predictive performance (AGC: R^2^ = 0.91, RMSE = 12.61 t hm^-2^; BGC: R^2^ = 0.95, RMSE = 3.94 t hm^-2^). However, for species combinations that included *B. gymnorrhiza*, both univariate and multivariate models resulted in significant errors (R^2^ < 0.5). Consequently, in this study, *B. gymnorrhiza* was treated individually, and its carbon stock was estimated using a multivariate model. Meanwhile, the univariate model for the *R. stylosa*-*A. marina* combination outperformed models developed for other combinations that included *R. stylosa* and *A. marina*.

**Fig 4:**
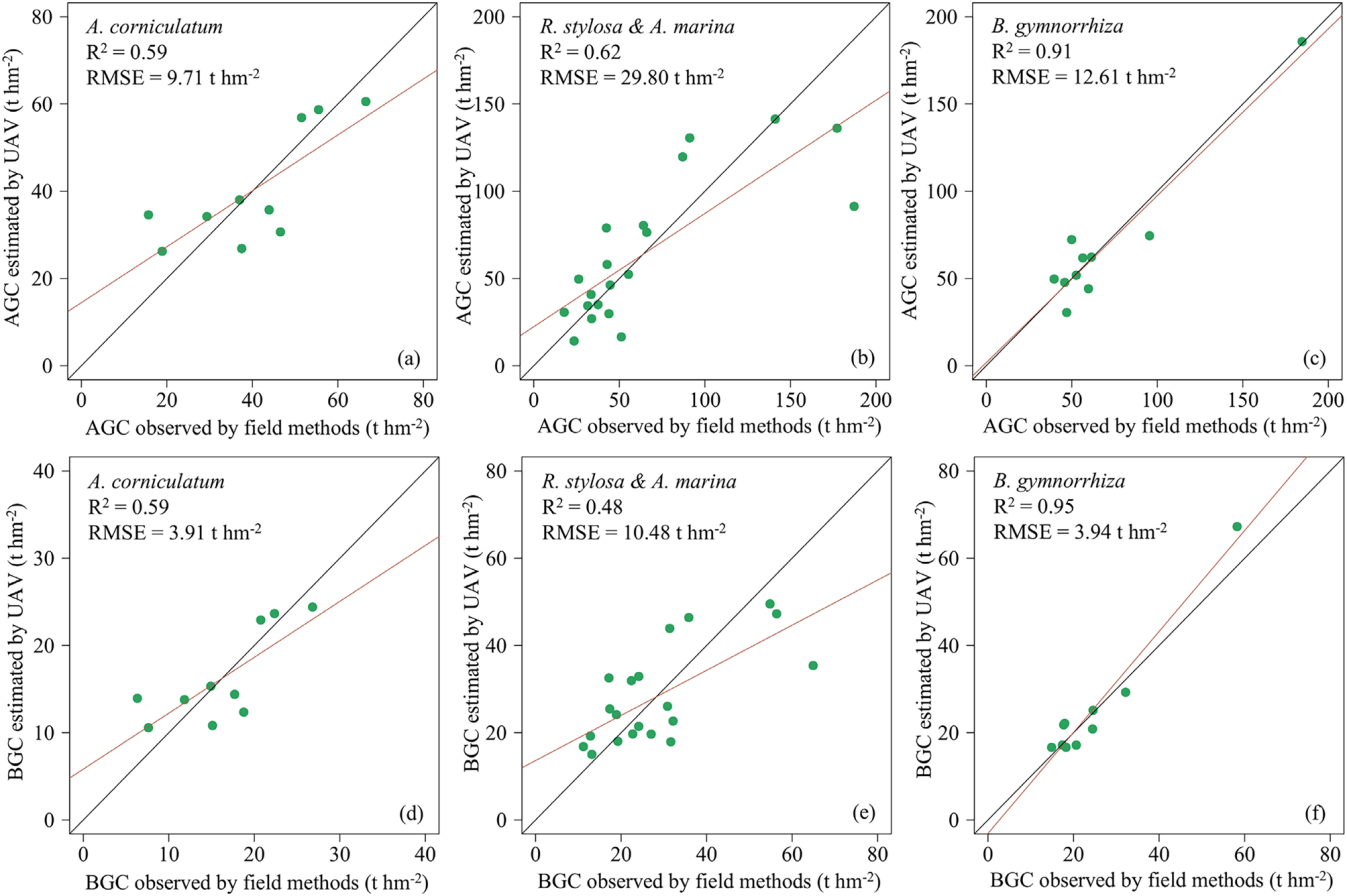
Carbon stock scatterplots of UAV predictions versus ground measurements based on leave-one-out-cross-validation. (**a**) is the model of A. corniculatum AGC; (**b**) is the model of R. stylosa - A. marina AGC; (**c**) is the model of B. gymnorrhiza AGC; (**d**) is the model of A. corniculatum BGC; (**e**) is the model of R. stylosa - A. marina BGC; (**f**) is the model of B. gymnorrhiza BGC. The red line indicates the scatter-fitted line between the UAV and ground results, and the diagonal black line represents the 1:1 relationship.

Over all, by comparing R^2^ and RMSE values for both univariate and multivariate models, we identified the best-performing models for estimating carbon stocks across all mangrove species in the study area (Fig. 4). The optimal above-ground carbon stock (AGC) models are as follows:

- *A. corniculatum*: AGC = 20.07RE_Mean - 464.53
- *B. gymnorrhiza*: AGC = 293.07B_Var - 31.21R_Con + 17.14
- *R. stylosa*-*A. marina*: AGC = 33.50H_Mean - 28.49

For below-ground carbon stock (BGC), the optimal models are:

- *A. Corniculatum*: BGC = 8.09RE_Mean - 187.21
- *B. Gymnorrhiza*: BGC = 95.55B_Var + 241.071NIR_Hom - 20.18B_Con - 54.10
- *R. stylosa*-*A. marina*: BGC = 9.20H_Mean + 2.75

The results showed that integrating spectral, textural, and structural features is helpful for species-level mangrove carbon stock estimation, and that the vegetation features contributing most to carbon estimation vary among mangrove species.

### Spatial distribution of mangrove carbon stock

**Fig 5:**
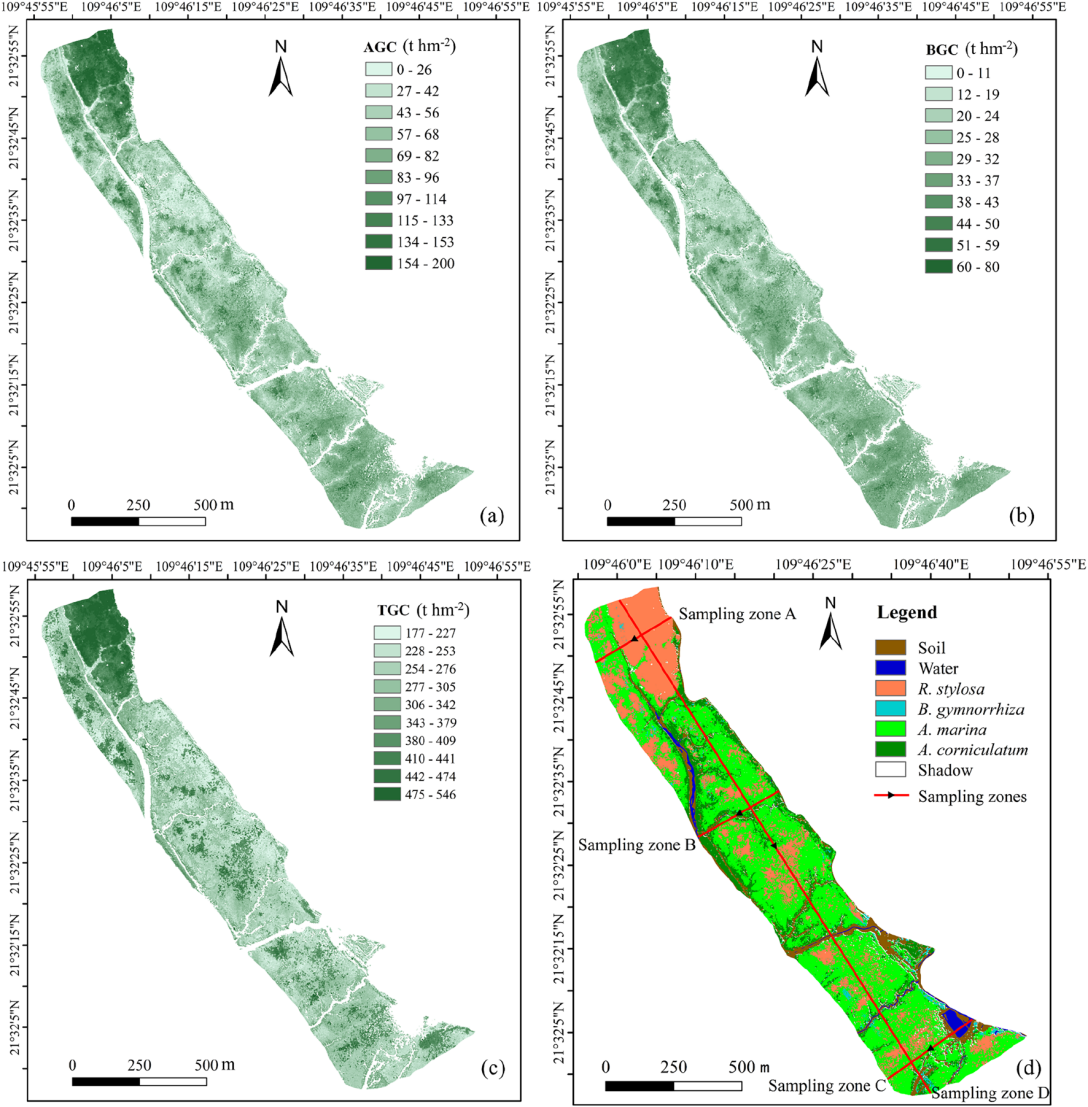
Spatial distribution of mangrove carbon stocks in the study area. (**a-c**) are above-ground carbon stock, below-ground carbon stock, and total carbon stock, respectively. The red lines on the orthomosaic map indicate the selected sampling zones (**d**), and their arrows indicate the sampling directions.

Based on the optimal species level models for mangrove carbon stock estimation, the AGC, BGC, and total carbon stocks (TGC) were calculated and their spatial distributions are shown in Fig. 5. The soil carbon stocks used for *A. marina*, *R. stylosa*, *A. corniculatum*, and *B. gymnorrhiza* were 177.37 t hm^-2^, 281.14 t hm^-2^, 236.18 t hm^-2^, and 257.67 t hm^-2^, respectively^55,56^.

**Fig 6:**
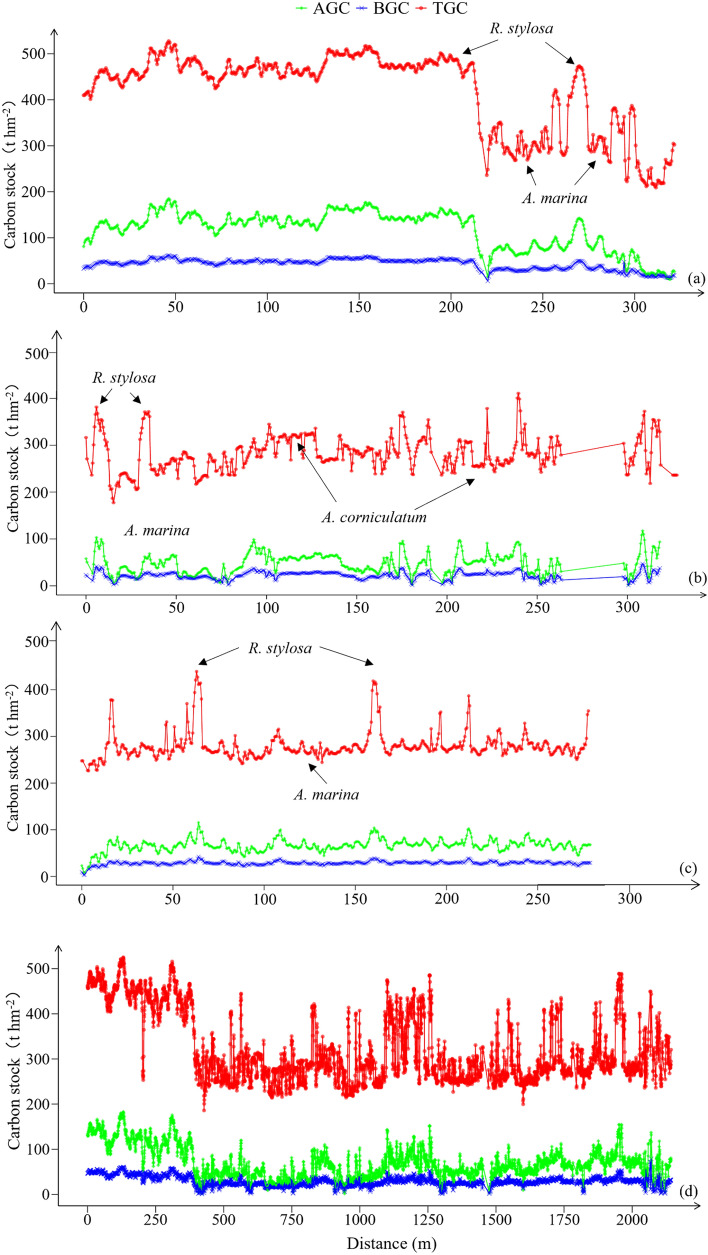
The carbon stock statistics of the typical sample zones. (**a-d**) are the above-ground carbon stock, below-ground carbon stock, and total carbon stock of **A**, **B**, **C** and **D** sample zones, respectively. “Distance” represents the distance along the arrow direction in the sampling zones from the starting point.

Higher carbon stocks were concentrated in the northwest of the study area, with smaller areas of high carbon stocks scattered elsewhere, mainly associated with the distribution of *R. stylosa*. In sample zone A, which is perpendicular to the coastline, AGC declined from 100–200 t hm⁻^2^ near the shore covered by *R. stylosa* to 60–80 t hm⁻^2^ further inland covered by *A. marina*, following the transition from high to low tidal zones. BGC and TGC exhibited similar decreasing trends (Fig. 6a). Sample zone B, located along the river, was predominantly occupied by *A. corniculatum*, with *A. marina* and *R. stylosa* present in smaller proportions. Carbon stock fluctuations were largely influenced by *A. marina* and *R. stylosa*, whereas *A. corniculatum* contributed to more stable carbon stock values (Fig. 6b). In sample zone C, which passes through a fish pond, *A. marina* was the dominant species, with smaller portions of *R. stylosa* and *A. corniculatum*. Here, carbon stock fluctuations were mainly driven by *R. stylosa*, while *A. marina* contributed to relatively stable values (Fig. 6c). The northwest part of the study area is located at the mouth of the Yingluo Bay estuary, where carbon stocks gradually decrease along the sample zone D (Fig. 6d).

**Fig 7:**
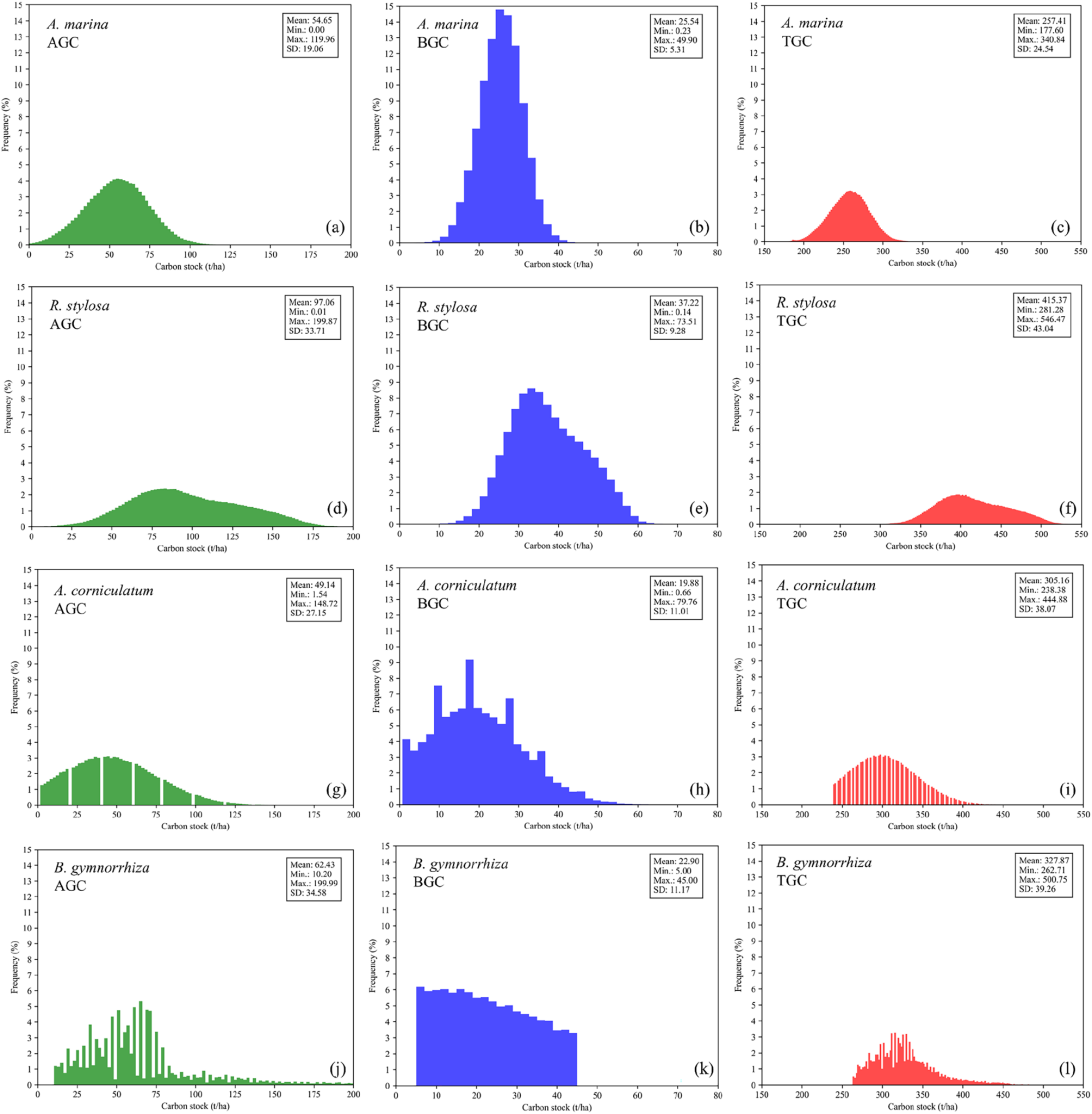
Distribution of above-ground carbon stock, below-ground carbon stock and total carbon stock of four mangrove species in the study area.

**Fig 8:**
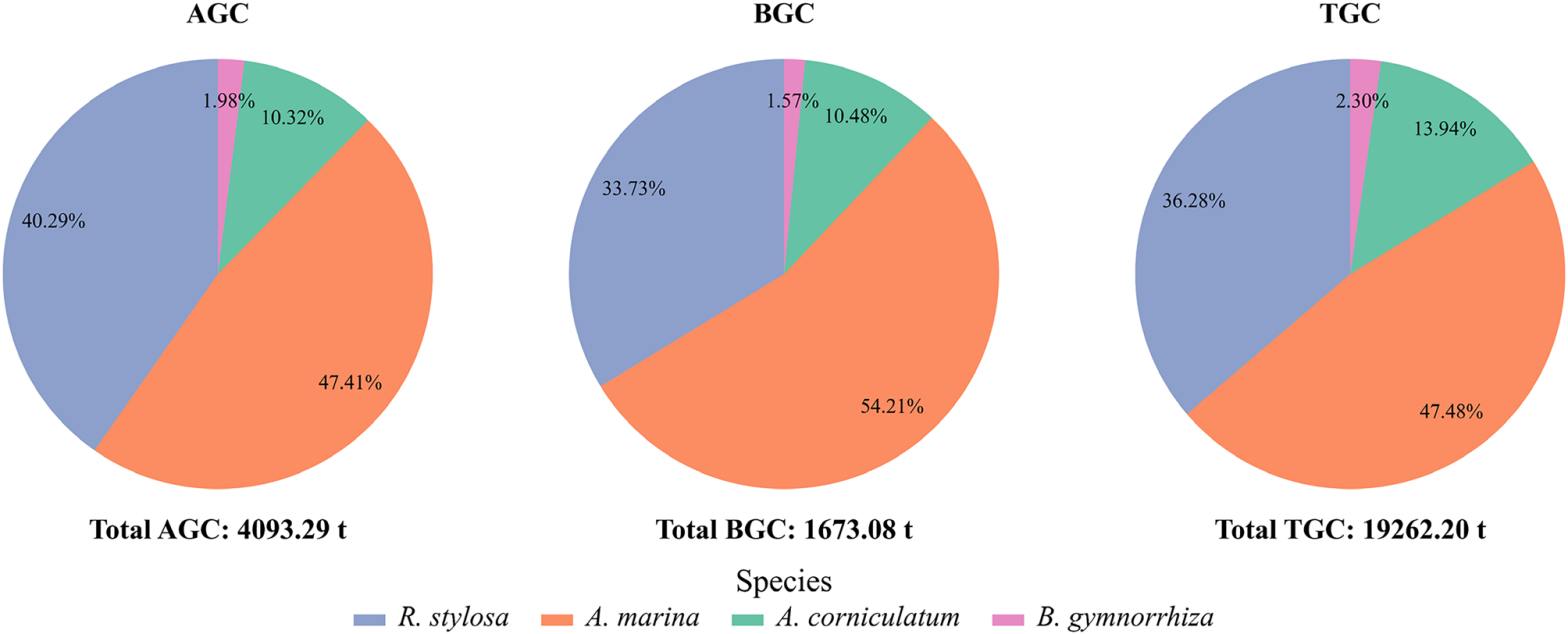
Distribution statistics of vegetation carbon stock of four mangroves.

The carbon stock distribution in the study area is shown in Fig. 7. The above-ground carbon stock is primarily distributed within the range of 40-70 t hm^-2^ for *A. marina*, 65-130 t hm^-2^ for *R. stylosa,* 20-70 t hm^-2^ for *A. corniculatum,* 30-80 t hm^-2^ for *B. gymnorrhiza,* while the below-ground carbon stock is mainly distributed within the range of 20-30 t hm^-2^ for *A. marina,* 25-50 t hm^-2^ for *R. stylosa,* 10-30 t hm^-2^ for *A. corniculatum,* 5-30 t hm^-2^ for *B. gymnorrhiza*. The average AGC for *A. marina*, *R. stylosa*, *A. corniculatum*, and *B. gymnorrhiza* is 54.65, 97.06, 49.14, and 62.43 t hm^-2^, respectively. The average BGC for *A. marina*, *R. stylosa*, *A. corniculatum*, and *B. gymnorrhiza* is 25.54, 37.22, 19.88, and 22.90 t hm^-2^, respectively. The proportions of AGC for *A. marina*, *R. stylosa*, *A. corniculatum*, and *B. gymnorrhiza* relative to the total AGC are 47.4%, 40.3%, 10.3%, 2.0%, respectively. The proportions of BGC for *A. marina*, *R. stylosa*, *A. corniculatum*, and *B. gymnorrhiza* relative to the total BGC are 54.2%, 33.7%, 10.5%, 1.6%, respectively. The total carbon stocks are 9146.12, 6988.31, 2685.38, and 442.39 t, respectively (Fig. 8).

## Discussion

### Advantage of the fusion of spectral and structural features for mangrove species identification

The identification of mangrove species in the study area relies heavily on the fusion of spectral and structural features derived from UAV multispectral technology. Among these, spectral features proved most significant, with vegetation indices such as NDVI, RVI, VREI, GNDVI, and SR, derived from the R, G, NIR, and RE bands, playing a central role (Fig. S2).

RE and NIR bands captured by multispectral sensors are essential for distinguishing mangrove species^57,58^. Visible light alone cannot reliably distinguish the 4 mangrove species in the study area. All species exhibited the highest reflectance in the G band and the lowest in the B band. *B. gymnorrhiza* showed the highest R and G band reflectance, while *R. stylosa* had the lowest in these bands. *A. marina* displayed the highest B band reflectance, with *A. corniculatum* and *A. marina* showing intermediate, similar R and G band values^59^. In the visible spectrum (400–700 nm), reflectance is dominated by chlorophyll a and b absorption^60^, while in the NIR range (700–1300 nm), leaf water content drives reflectance^61^. As a result, the similar relative chlorophyll content (SPAD values) of the 4 species complicates identification: *R. stylosa* (65.9) and *B. gymnorrhiza* (62.9) have similar values, as followed by *A. marina* (51.9) and *A. corniculatum* (48.6), with SPAD positively correlated to plant height and basal diameter^62,63^. The inclusion of NIR and RE bands enhanced the discrimination between *R. stylosa* and *B. gymnorrhiza,* as well as between *A. marina* and *A. corniculatum.*

Structural features, such as tree height, further improved identification accuracy. *B. gymnorrhiza* and *R. stylosa* differ in height from *A. marina* and *A. corniculatum*, allowing the integration of spectral and structural features to increase mapping precision, particularly for *B. gymnorrhiza* and *A. corniculatum*, while slightly enhancing soil classification.

Textural features have been reported to improve mangrove species classification in some studies^64,65^. However, they were less effective for species identification in this study. Texture, which describes image variability, relies on regular images or uniform regions (such as small patches) and do not take color information into account^66^. Canopy textural information depends on canopy shadows, leaf traits (e.g., size and reflectivity), and branching patterns^67^. Among the study species, *R. stylosa* and *B. gymnorrhiza* have large, elongated leaves (*R. stylosa* slightly larger), while *A. corniculatum* and *A. marina* have similar elliptical leaves (*A. corniculatum* slightly larger). These subtle differences, combined with complex canopy structures, limit the utility of textural features for distinguishing these species.

### Advantage of the fusion of spectral, structural and textural features for species-level mangrove carbon stock estimation

The fusion of spectral, structural, and textural features offered significant advantages for estimating mangrove carbon stocks at species level. The inversion of mangrove carbon stock typically uses spectral features, particularly vegetation indices, as input variables^23,68,69^, with fewer studies integrating textural and structural variables concurrently. Hidayatullah et al.^15^ employed GEOBIA with WorldView-2 imagery to map mangrove species and estimate AGC using vegetation indices. Indices incorporating R, RE, and NIR bands effectively predicted species-specific AGC, consistent with our results; however, textural and structural features were not considered in their approach.

Incorporating structural features with spectral information has been proved to provide better biomass estimates in forest landscapes with complex species compositions^70^. Previous studies^27,71^ demonstrated that UAV LiDAR–derived structural features, particularly canopy height, enhance mangrove AGC estimation when height variation is pronounced, which is consistent with the effectiveness of H_Mean for predicting AGC and BGC in mixed-species stands (e.g., *R. stylosa* with *A. marina* or *A. corniculatum*) in our study. However, textural features were not considered in these studies. Textural features offer predictive power for carbon stocks, complementing spectral and structural data. Wang et al.^72^ and Li et al.^6^ identified multiple textural variables as effective AGC predictors, while Wang et al.^32^ further showed that integrating spectral and textural features improved both AGC and BGC estimation. Consistent with these findings, our results indicate that textural features derived from the R, B, and RE bands were effective for AGC estimation of 4 mangrove species, whereas textural features from the B, RE, and NIR bands were key for BGC estimation.

The advantages of triple-feature fusion are largely enabled by UAV multispectral technology, which provides high spatial resolution and allows the simultaneous extraction of spectral, structural, and textural features. This approach supports a more comprehensive and accurate assessment of mangrove carbon stocks at species level than approaches relying on a single category of features.

### Influence of species on carbon stocks and its estimation

The results revealed significant interspecific variations in mangrove carbon stocks, primarily driven by differences in dominant species within each vegetation type and their structural characteristics, particularly tree height and basal area^73^. In our study, *R. stylosa* exhibited substantially higher mean AGC and BGC values (97.06 t/hm^2^ and 37.22 t/hm^2^, respectively) than other species, attributable to its tall stature, robust trunk, and extensive branching system. Previous studies have shown that arborescent mangroves generally exhibit higher growth rates and accumulate more biomass than shrub species^74,75^. Carbon accumulation increases with tree size, as large trees account for most biomass variation in forests and maintain high carbon sequestration capacity even at maturity^76,77^. This size-dependent effect explains the concentration of high carbon stocks in *R. stylosa*-dominated areas, as well as higher mean AGC and BGC of *B. gymnorrhiza* relative to *A. marina* and *A. corniculatum* in our study site. Tree density alone does not necessarily correlate positively with carbon stock, as shrub forests are typically denser but structurally less developed than low-density mature arborescent forests^78^. Accordingly, *A. marina* and *A. corniculatum* exhibited lower mean AGC and BGC values than *R. stylosa* and *B. gymnorrhiza* despite their higher stand densities. Beyond vegetation biomass, species composition and stand age further modulate ecosystem-level carbon storage through their effects on litter input, root turnover, and soil organic carbon formation^55,79–81^. Our study area exhibited a landward to seaward age gradient, with decreasing tree age corresponding to reduced vegetation carbon storage in younger mangroves compared to mature stands.

Species also influence carbon stock estimation methodologies, highlighting the necessity of developing species-specific carbon estimation models. Li et al.^6^ demonstrated that species type was the most influential variable in UAV-based mangrove AGC estimation, underscoring the importance of species-level analysis, although species-specific models were not developed. In contrast, our study established species-specific carbon stock estimation models using species-specific allometric equations as reference values. Significant discrepancies have been reported between generic and species-specific allometric equations in mangrove AGC estimation, with species-specific equations producing more realistic AGC ranges^7,82^. Moreover, the applicability of estimation models and optimal predictive variables varies considerably among mangrove species^27,70^. Consistent with this, our results showed that the most effective vegetation features differed by species: structural feature H_Mean proved most effective for *R. stylosa* and *A. marina*, consistent with findings by Salum et al.^83^ and Jones et al.^84^; the textural feature RE_Mean was optimal for *A. corniculatum*, and distinct combinations of textural features for *B. gymnorrhiza* in AGC and BGC estimation. The biophysical characteristics of each mangrove species and the vegetation features used as predictive variables collectively determined model performance, ultimately influencing species-level carbon stock estimates^15^.

### Application and limitations

This study effectively utilized UAV multispectral technology to identify mangrove species and develop species-level carbon stock models for mangrove carbon stock estimation. The methodology was applied to the mangroves located along the midstream coast of Yingluo Bay’s estuarine mangroves. Our estimation results aligned closely with ground-based surveys of the broader Yingluo Bay area by Wang et al.^85^ and Yu et al.^81^ (Table S11). This consistency validates the model as a reliable tool for mangrove carbon stock assessment.

This study has several limitations. First, UAV multispectral imagery was resampled from the original 0.045 m resolution to 0.53 m to reduce data volume and match field plot scales; while appropriate for this study, the resampling inherently affects feature extraction and may influence classification and carbon estimation results. Second, species classification was performed using the traditional maximum likelihood classifier, which is interpretable but may underperform compared with modern machine-learning approaches such as random forest^86^, XGBoost^87^ or deep learning algorithms^36^; future studies could incorporate advanced classifiers to improve accuracy and reproducibility. Third, the reliance on linear regression may oversimplify ecological interactions, and the study only captured spatial patterns at a single time point, lacking temporal insights such as restoration activities, natural disturbances, and successional changes; future research could explore nonlinear or machine-learning models and multi-temporal UAV observations to enhance carbon stock assessment and capture temporal dynamics^88,89^. Finally, extending the framework to estimate carbon stocks at the individual plant scale could provide finer-resolution insights for mangrove carbon assessments.

## Conclusion

Our study highlights the significant influence of interspecific differences on mangrove carbon stocks and their estimation, and establishes a framework for species-level mangrove carbon stock estimation using UAV multispectral technology. Different mangrove species exhibit distinct vegetation features for carbon stock modeling, and the fusion of spectral, textural, and structural vegetation features improved mangrove species mapping and species-level carbon estimation. These findings suggest that effective monitoring, conservation, and restoration of mangrove carbon stocks should explicitly consider species composition, enabling more precise carbon accounting and informed decision-making in blue carbon management.

Future research should extend the proposed methodology to diverse mangrove communities and explore additional modeling approaches to further refine species-level carbon stock estimations.

## Supplementary Information

Supplementary Information.

## Data Availability

Data provided in the manuscript and supplementary file. Further details, the corresponding author can provide the data used and analyzed in this study upon request.

## References

[1] Mcleod, E. et al. A blueprint for blue carbon: Toward an improved understanding of the role of vegetated coastal habitats in sequestering CO_2_. *Front. Ecol. Environ.* **9**, 552–560. 10.1890/110004 (2011).

[2] Madhavan, C., Meera, S. P. & Kumar, A. Anatomical adaptations of mangroves to the intertidal environment and their dynamic responses to various stresses. *Biol. Rev.*10.1111/brv.13172 (2024). 39654142 10.1111/brv.13172

[3] Donato, D. C. et al. Mangroves among the most carbon-rich forests in the tropics. *Nat. Geosci.* **4**, 293–297. 10.1038/ngeo1123 (2011).

[4] Bai, J. et al. Mangrove diversity enhances plant biomass production and carbon storage in Hainan island, China. *Funct. Ecol.* **35**(3), 774–786. 10.1111/1365-2435.13753 (2021).

[5] Sitthi, A., Pimple, U., Piponiot, C. & Gond, V. Assessing the effectiveness of mangrove rehabilitation using above-ground biomass and structural diversity. *Sci. Rep.* **15**, 7839. 10.1038/s41598-025-92514-7 (2025). 40050393 10.1038/s41598-025-92514-7 PMC11885591

[6] Li, Z. et al. Remote estimation of mangrove aboveground carbon stock at the species level using a low-cost unmanned aerial vehicle system. *Remote Sens.* **11**, 1018. 10.3390/rs11091018 (2019).

[7] Zhang, R. & Fan, J. Classification and carbon-stock estimation of mangroves in dongzhaigang based on multi-source remote sensing data using google earth engine. *Remote Sens.* **17**(6), 964. 10.3390/rs17060964 (2025).

[8] Rao, K., Ramanathan, A. L. & Raju, N. J. Assessment of blue carbon stock of Coringa mangroves: Climate change perspective. *J. Clim. Change* **8**, 41–58. 10.3233/JCC220013 (2022).

[9] Binh, N. A. et al. Monitoring mangrove traits through optical Earth observation: Towards spatio-temporal scalability using cloud-based Sentinel-2 continuous time series. *ISPRS J. Photogramm. Remote Sens.* **214**, 135–152. 10.1016/j.isprsjprs.2024.06.007 (2024).

[10] Roy, A. D. et al. Remote sensing-based mangrove blue carbon assessment in the Asia-Pacific: A systematic review. *Sci. Total Environ.* **938**, 173270. 10.1016/j.scitotenv.2024.173270 (2024). 38772491 10.1016/j.scitotenv.2024.173270

[11] Ruwaimana, M. et al. The advantages of using drones over space-borne imagery in the mapping of mangrove forests. *Plos One* **13**, 0200288. 10.1371/journal.pone.0200288 (2018). 10.1371/journal.pone.0200288 PMC6051606 30020959

[12] Lu, D. et al. A survey of remote sensing-based aboveground biomass estimation methods in forest ecosystems. *Int. J. Dig. Earth* **9**, 63–105. 10.1080/17538947.2014.990526 (2014).

[13] Lu, D. The potential and challenge of remote sensing-based biomass estimation. *Int. J. Remote Sens.* **27**, 1297–1328. 10.1080/01431160500486732 (2007).

[14] Zhu, Y. et al. Estimating and mapping mangrove biomass dynamic change using WorldView-2 images and digital surface models. *IEEE J. Sel. Top. Appl. Earth Obs. Remote Sens.* **13**, 2123–2134. 10.1109/JSTARS.2020.2989500 (2020).

[15] Hidayatullah, M. F., Kamal, M. & Wicaksono, P. Species-based aboveground mangrove carbon stock estimation using WorldView-2 image data. *Remote Sens. Appl.: Soc. Environ.* **30**, 100959. 10.1016/j.rsase.2023.100959 (2023).

[16] Navarro, A. et al. The application of unmanned aerial vehicles (UAVs) to estimate above-ground biomass of mangrove ecosystems. *Remote Sens. Environ.* **242**, 111747. 10.1016/j.rse.2020.111747 (2020).

[17] Tian, Y. et al. Aboveground mangrove biomass estimation in Beibu Gulf using machine learning and UAV remote sensing. *Sci. Total Environ.* **781**, 146816. 10.1016/j.scitotenv.2021.146816 (2021).

[18] Tait, L. et al. Unmanned aerial vehicles (UAVs) for monitoring macroalgal biodiversity: Comparison of RGB and multispectral imaging sensors for biodiversity assessments. *Remote Sens.* **11**, 2332. 10.3390/rs11192332 (2019).

[19] Cao, J. et al. Object-based mangrove species classification using unmanned aerial vehicle hyperspectral images and digital surface models. *Remote Sens.* **10**, 89. 10.3390/rs10010089 (2018).

[20] Yang, Y. et al. Fine-scale mangrove species classification based on uav multispectral and hyperspectral remote sensing using machine learning. *Remote Sens.* **16**(16), 3093. 10.3390/rs16163093 (2024).

[21] Luo, S. et al. Fusion of airborne LiDAR data and hyperspectral imagery for aboveground and belowground forest biomass estimation. *Ecol. Ind.* **73**, 378–387. 10.1016/j.ecolind.2016.10.001 (2017).

[22] De Almeida, D. R. A. et al. Monitoring restored tropical forest diversity and structure through UAV-borne hyperspectral and LiDAR fusion. *Remote Sens. Environ.* **264**, 112582. 10.1016/j.rse.2021.112582 (2021).

[23] Xiang, H. et al. Community identification and carbon storage monitoring of Heritiera littoralis with UAV hyperspectral imaging. *Ecol. Ind.* **167**, 112653. 10.1016/j.ecolind.2024.112653 (2024).

[24] Tao, H. et al. Estimation of crop growth parameters using UAV-based hyperspectral remote sensing data. *Sensors* **20**(5), 1296. 10.3390/s20051296 (2020). 32120958 10.3390/s20051296 PMC7085721

[25] Dalla Corte, A. P. et al. Measuring individual tree diameter and height using GatorEye high-density UAV-LiDAR in an integrated crop-livestock-forest system. *Remote Sens.* **12**, 863. 10.3390/rs12050863 (2020).

[26] Salum, R. B. et al. Improving mangrove above-ground biomass estimates using LiDAR. *Estuarine, Coastal Shelf Sci.* **236**, 106585. 10.1016/j.ecss.2020.106585 (2020).

[27] Li, S. et al. Estimation of aboveground biomass of different vegetation types in mangrove forests based on UAV remote sensing. *Sustain. Horiz.* **11**, 100100. 10.1016/j.horiz.2024.100100 (2024).

[28] Yin, D., Wang, L., Lu, Y. & Shi, C. Mangrove tree height growth monitoring from multi-temporal UAV-LiDAR. *Remote Sens. Environ.* **303**, 114002. 10.1016/j.rse.2024.114002 (2024).

[29] Nesha, M. K., Hussin, Y. A., van Leeuwen, L. M. & Sulistioadi, Y. B. Modeling and mapping aboveground biomass of the restored mangroves using ALOS-2 PALSAR-2 in East Kalimantan, Indonesia. *Int. J. Appl. Earth Obs. Geoinform.* **91**, 102158. 10.1016/j.jag.2020.102158 (2020).

[30] Hickey, S. M., Callow, N. J., Phinn, S., Lovelock, C. E. & Duarte, C. M. Spatial complexities in aboveground carbon stocks of a semi-arid mangrove community: A remote sensing height-biomass-carbon approach. *Estuarine, Coastal Shelf Sci.* **200**, 194–201. 10.1016/j.ecss.2017.11.004 (2018).

[31] Chen, R., Zhang, R., Zhao, C., Wang, Z. & Jia, M. High-resolution mapping of mangrove species height in fujian zhangjiangkou national mangrove nature reserve combined GF-2, GF-3, and UAV-LiDAR. *Remote Sens.* **15**(24), 5645. 10.3390/rs15245645 (2023).

[32] Wang, Z. et al. Regional mangrove vegetation carbon stocks predicted integrating UAV-LiDAR and satellite data. *J. Environ. Manag.* **368**, 122101. 10.1016/j.jenvman.2024.122101 (2024). 10.1016/j.jenvman.2024.122101 39173298

[33] Yin, D. & Wang, L. Individual mangrove tree measurement using UAV-based LiDAR data: Possibilities and challenges. *Remote Sens. Environ.* **223**, 34–49. 10.1016/j.rse.2018.12.034 (2019).

[34] Jain, K. & Pandey, A. Calibration of satellite imagery with multispectral UAV imagery. *J. Indian Soc. Remote Sens.* **49**, 479–490. 10.1007/s12524-020-01251-z (2020).

[35] Fu, B. et al. Comparison of RFE-DL and stacking ensemble learning algorithms for classifying mangrove species on UAV multispectral images. *Int. J. Appl. Earth Obs. Geoinform.* **112**, 102890. 10.1016/j.jag.2022.102890 (2022).

[36] Li, Y. et al. Comparison of different transfer learning methods for classification of mangrove communities using MCCUNet and UAV multispectral images. *Remote Sens.* **14**, 5533. 10.3390/rs14215533 (2022).

[37] Chen, X. et al. An approach for detecting mangrove areas and mapping species using multispectral drone imagery and deep learning. *Sensors* **25**(8), 2540. 10.3390/s25082540 (2025). 40285231 10.3390/s25082540 PMC12031454

[38] Kolanuvada, S. R. & Ilango, K. K. Automatic extraction of tree crown for the estimation of biomass from UAV imagery using neural networks. *J. Indian Soc. Remote Sens.* **49**, 651–658. 10.1007/s12524-020-01242-0 (2021).

[39] Lian, X. et al. Biomass calculations of individual trees based on unmanned aerial vehicle multispectral imagery and laser scanning combined with terrestrial laser scanning in complex stands. *Remote Sens.* **14**, 4715. 10.3390/rs14194715 (2022).

[40] Tan, H. et al. Improved estimation of aboveground biomass in rubber plantations using deep learning on UAV multispectral imagery. *Drones* **9**(1), 32. 10.3390/drones9010032 (2025).

[41] Zhao, D. et al. Spatiotemporal dynamics and geo-environmental factors influencing mangrove gross primary productivity during 2000–2020 in Gaoqiao Mangrove Reserve, China. *For. Ecosyst.* **10**, 100137. 10.1016/j.fecs.2023.100137 (2023).

[42] Cao Q. The estimation of mangrove biomass and carbon storageusing remote sensing data in Beibu Gulf coast. Chinese Academy of Forestry. (2010).

[43] Tran, T. V., Reef, R. & Zhu, X. A review of spectral indices for mangrove remote sensing. *Remote Sens.* **14**, 4868. 10.3390/rs14194868 (2022).

[44] Haralick, R. M., Shanmugam, K. & Dinstein, I. H. Textural features for image classification. *IEEE Trans. Syst., Man, Cybern.* **6**, 610–621. 10.1109/TSMC.1973.4309314 (2007).

[45] Huang, X., Zhang, L. & Wang, L. Evaluation of morphological texture features for mangrove forest mapping and species discrimination using multispectral IKONOS imagery. *IEEE Geosci. Remote Sens. Lett.* **6**, 393–397. 10.1109/LGRS.2009.2014398 (2009).

[46] Eckert, S. Improved forest biomass and carbon estimations using texture measures from WorldView-2 satellite data. *Remote Sens.* **4**(4), 810–829. 10.3390/rs4040810 (2012).

[47] Li, H., Han, Y. & Chen, J. Combination of google earth imagery and Sentinel-2 data for mangrove species mapping. *J. Appl. Remote Sens.* **14**, 010501. 10.1117/1.JRS.14.010501 (2020).

[48] Li, Q., Wong, F. K. K. & Fung, T. Classification of mangrove species using combined WordView-3 and LiDAR data in Mai Po Nature Reserve, Hong Kong. *Remote Sens.* **11**(18), 2114. 10.3390/rs11182114 (2019).

[49] Simpson, G. et al. Species-level classification of peatland vegetation using ultra-high-resolution UAV imagery. *Drones* **8**(3), 97. 10.3390/drones8030097 (2024).

[50] Bhadra, T. et al. Monitoring the mangroves of Indian Sundarbans using geospatial techniques. *Int. Arch. Photogramm., Remote Sens. Spatial Inform. Sci.* **48**, 405–412. 10.5194/isprs-archives-XLVIII-1-W2-2023-405-2023 (2023).

[51] Gao, L., Chai, G. & Zhang, X. Above-ground biomass estimation of plantation with different tree species using airborne LiDAR and hyperspectral data. *Remote Sens.* **14**, 2568. 10.3390/rs14112568 (2022).

[52] Pan, H. L., Huang, C. M. & Huang, C. Y. Mapping aboveground carbon density of subtropical subalpine dwarf bamboo (Yushania niitakayamensis) vegetation using UAV-lidar. *Int. J. Appl. Earth Obs. Geoinform.* **123**, 103487. 10.1016/j.jag.2023.103487 (2023).

[53] Niu, X. et al. Estimation of coastal wetland vegetation aboveground biomass by integrating UAV and satellite remote sensing data. *Remote Sens.* **16**(15), 2760. 10.3390/rs16152760 (2024).

[54] Shao, J. Linear model selection by cross-validation. *J. Am. Stat. Assoc.* **88**, 486–494. 10.1080/01621459.1993.10476299 (1993).

[55] Wang, G., Guan, D., Peart, M. R., Chen, Y. & Peng, Y. Ecosystem carbon stocks of mangrove forest in Yingluo Bay, Guangdong Province of South China. *For. Ecol. Manag.* **310**, 539–546. 10.1016/j.foreco.2013.08.045 (2013).

[56] Wang, G. et al. Changes in mangrove community structures affecting sediment carbon content in Yingluo Bay of South China. *Mar. Pollut. Bull.* **149**, 110581. 10.1016/j.marpolbul.2019.110581 (2019). 31550580 10.1016/j.marpolbul.2019.110581

[57] Simarmata, N. et al. Mangrove ecosystem species mapping from integrated Sentinel-2 imagery and field spectral data using random forest algorithm. *J. Appl. Remote Sens.* **18**(1), 014509–014509. 10.1117/1.JRS.18.014509 (2024).

[58] Chen, Z., Zhang, M., Zhang, H. & Liu, Y. Mapping mangrove using a red-edge mangrove index (REMI) based on Sentinel-2 multispectral images. *IEEE Trans. Geosci. Remote Sens.* **61**, 1–11. 10.1109/TGRS.2023.3323741 (2023).

[59] Zhang, P., Shang, X. & Wu, Z. Morphological characteristics of leaves and estimation of relative chlorophyll content in five species of mangrove based on image processing technology. *Chin. J. Trop. Crops* **41**(3), 496–503 (2020).

[60] Wolff, F., Lorenz, S., Korpelainen, P., Eltner, A. & Kumpula, T. UAV and field hyperspectral imaging for Sphagnum discrimination and vegetation modelling in Finnish aapa mires. *Int. J. Appl. Earth Obs. Geoinform.* **134**, 104201. 10.1016/j.jag.2024.104201 (2024).

[61] Gan, Y., Wang, Q. & Song, G. Structural complexity significantly impacts canopy reflectance simulations as revealed from reconstructed and sentinel-2-monitored scenes in a temperate deciduous forest. *Remote Sens.* **16**(22), 4296. 10.3390/rs16224296 (2024).

[62] Zhang, W., Chen, Y., Shang, X., Xu, F. & Wu, Z. Comparison on SPAD values of leaves in five species of mangroves. *Subtrop. Plant Sci.* **45**(03), 212 (2016).

[63] Jiang, D. et al. A trade-off between leaf carbon economics and plant size among mangrove species in dongzhaigang, China. *Ecol. Evol.* **14**(11), e70559. 10.1002/ece3.70559 (2024). 39563707 10.1002/ece3.70559 PMC11576130

[64] Wang, T., Zhang, H., Lin, H. & Fang, C. Textural–spectral feature-based species classification of mangroves in Mai Po Nature Reserve from Worldview-3 imagery. *Remote Sens.* **8**(1), 24. 10.3390/rs8010024 (2015).

[65] Sun, Y. et al. Species classification and carbon stock assessment of mangroves in Qi’ao Island with Worldview-3 imagery. *Forests* **14**(12), 2356. 10.3390/f14122356 (2023).

[66] Liu, Y., Zhang, D., Lu, G. Ma, W. Y. Study on texture feature extraction in region-based image retrieval system. *In 2006 12th International Multi-Media Modelling Conference*, 8-pp. IEEE 10.1109/MMMC.2006.1651329 (2006).

[67] Franklin, S. E., Hall, R. J., Moskal, L. M., Maudie, A. J. & Lavigne, M. B. Incorporating texture into classification of forest species composition from airborne multispectral images. *Int. J. Remote Sens.* **21**, 61–79. 10.1080/014311600210993 (2010).

[68] Purnamasari, E., Kamal, M. & Wicaksono, P. Comparison of vegetation indices for estimating above-ground mangrove carbon stocks using PlanetScope image. *Reg. Stud. Mar. Sci.* **44**, 101730. 10.1016/j.rsma.2021.101730 (2021).

[69] Mariano Neto, M., da Silva, J. B. & de Brito, H. C. Carbon stock estimation in a Brazilian mangrove using optical satellite data. *Environ. Monit. Assess.* **196**(1), 9. 10.1007/s10661-023-12151-3 (2024). 10.1007/s10661-023-12151-3 38049645

[70] Li, Q. et al. Tree-level carbon stock estimations across diverse species using multi-source remote sensing integration. *Comput. Electron. Agric.* **231**, 109904. 10.1016/j.compag.2025.109904 (2025).

[71] Wu, P. et al. Fine identification and biomass estimation of mangroves based on UAV multispectral and LiDAR. *Natl. Remote Sens. Bull* **26**, 1169–1181 (2022).

[72] Wang, M. et al. Potential of texture metrics derived from high-resolution Pleiades satellite data for quantifying aboveground carbon of Kandelia candel mangrove forests in Southeast China. *Wetlands Ecol. Manag.* **26**, 789–803. 10.1007/s11273-018-9610-2 (2018).

[73] Ruiz-Jaen, M. C. & Potvin, C. Tree diversity explains variation in ecosystem function in a neotropical forest in Panama. *Biotropica* **42**(6), 638–646. 10.1111/j.1744-7429.2010.00631.x (2010).

[74] Chen, S. et al. Higher soil organic carbon sequestration potential at a rehabilitated mangrove comprised of Aegiceras corniculatum compared to Kandelia obovata. *Sci. Total Environ.* **752**, 142279. 10.1016/j.scitotenv.2020.142279 (2021). 33207510 10.1016/j.scitotenv.2020.142279

[75] Kang, L. et al. Carbon storage potential and influencing factors of mangrove plantation in Kaozhouyang, Guangdong Province, South China. *Front. Mar. Sci.* **11**, 1439266. 10.3389/fmars.2024.1439266 (2025).

[76] Lv, Z., Duan, A. & Zhang, J. Influence of forest age, tree size, and climate factors on biomass and carbon storage allocation in Chinese fir forests. *Ecol. Ind.* **163**, 112096. 10.1016/j.ecolind.2024.112096 (2024).

[77] Stephenson, N. L. et al. Rate of tree carbon accumulation increases continuously with tree size. *Nature* **507**, 90–93. 10.1038/nature12914 (2014). 24429523 10.1038/nature12914

[78] Rani, V. et al. Carbon stock in biomass pool of fragmented mangrove habitats of Kochi, Southern India. *Environ. Sci. Pollut. Res.* **30**(43), 96746–96762. 10.1007/s11356-023-29069-5 (2023). 10.1007/s11356-023-29069-5 37581732

[79] Mukherjee, J., Ray, S. & Ghosh, P. B. A system dynamic modeling of carbon cycle from mangrove litter to the adjacent Hooghly estuary, India. *Ecol. Model.* **252**, 185–195. 10.1016/j.ecolmodel.2012.06.036 (2013).

[80] Wang, G. et al. Spatial patterns of biomass and soil attributes in an estuarine mangrove forest (Yingluo Bay, South China). *Eur. J. For. Res.* **133**, 993–1005. 10.1007/s10342-014-0817-3 (2014).

[81] Yu, C. et al. Development of ecosystem carbon stock with the progression of a natural mangrove forest in Yingluo Bay, China. *Plant Soil* **460**, 391–401. 10.1007/s11104-020-04819-3 (2021).

[82] Kamal, M., Hidayatullah, M. F., Mahyatar, P. & Ridha, S. M. Estimation of aboveground mangrove carbon stocks from WorldView-2 imagery based on generic and species-specific allometric equations. *Remote Sens. Appl.: Soc. Environ.* **26**, 100748. 10.1016/j.rsase.2022.100748 (2022).

[83] Salum, R. B., Robinson, S. A. & Rogers, K. A validated and accurate method for quantifying and extrapolating mangrove above-ground biomass using LiDAR data. *Remote Sens.* **13**(14), 2763. 10.3390/rs13142763 (2021).

[84] Jones, A. R. et al. Estimating mangrove tree biomass and carbon content: A comparison of forest inventory techniques and drone imagery. *Front. Mar. Sci.* **6**, 784. 10.3389/fmars.2019.00784 (2020).

[85] Wang, G., Guan, D., Xiao, L. & Peart, M. R. Ecosystem carbon storage affected by intertidal locations and climatic factors in three estuarine mangrove forests of South China. *Reg. Environ. Change* **19**, 1701–1712. 10.1007/s10113-019-01515-6 (2019).

[86] Miraki, M., Sohrabi, H. & Immitzer, M. Tree species mapping in mangrove ecosystems using UAV-RGB imagery and object-based image classification. *J. Indian Soc. Remote Sens.* **51**, 2095–2103. 10.1007/s12524-023-01752-7 (2023).

[87] Zhen, J. et al. Performance of xgboost ensemble learning algorithm for mangrove species classification with multisource spaceborne remote sensing data. *J. Remote Sens.* **4**, 0146. 10.34133/remotesensing.0146 (2024).

[88] Tunca, E., Köksal, E. S., Akay, H., Öztürk, E. & Taner, S. Ç. Novel machine learning framework for high-resolution sorghum biomass estimation using multi-temporal UAV imagery. *Int. J. Environ. Sci. Technol.* **22**, 13673–13688. 10.1007/s13762-025-06498-y (2025).

[89] Wei, L. et al. Wheat biomass, yield, and straw-grain ratio estimation from multi-temporal UAV-based RGB and multispectral images. *Biosyst. Eng.* **234**, 187–205. 10.1016/j.biosystemseng.2023.08.002 (2023).

