# Supplementary material for: Triple-feature fusion from UAV multispectral imagery enhances species-level mangrove carbon assessment
Source: Sci Rep. 2026 Mar 1;16:11494. doi: 10.1038/s41598-026-40303-1 (PMC13057202; doi:10.1038/s41598-026-40303-1)
Supplement: Supplementary file 1 — Supplementary Information. [file 41598_2026_40303_MOESM1_ESM.docx]

**Triple-Feature Fusion from UAV Multispectral Imagery Enhances Species-Level Mangrove Carbon Assessment**

Yu Chen^1,2^, Xiaoxue Shen^1,2^, ChunhuaYan^3,4^, Biqian Jiang^1,2^, Ruili Li^1,2*^, Minwei Chai^1,2^

1 *School of Environment and Energy, Shenzhen Graduate School, Peking University, Shenzhen 518055, China*

2 *Guangdong Mangrove Engineering Technology Research Center, Peking University, Shenzhen 518055, China*

3 *School of Ecology, Sun Yat-sen University, Guangzhou 510275, China*

4 *Shenzhen Campus of Sun Yat-sen University, Shenzhen 518107, China*

Correspondence:

Ruili Li, School of Environment and Energy, Shenzhen Graduate School of Peking University, Shenzhen

Email: liruili@pkusz.edu.cn

Phone: +86 755 26033141

**Supplementary** **material**

Table S1 Biomass allometric equations and carbon conversion coefficients of different mangrove species

| Species | Equation | Reference | Carbon conversion coefficients | |
| --- | --- | --- | --- | --- |
| *R. stylosa* | lg(AGB) = 2.465lg(DBH)-0.696 | [1] | 43.18% | [7] |
|  | lg(BGB) = 1.860lg(DBH)-0.583 |  |  |  |
| *A. marina* | AGB = 0.0543((D^2^)H)^1.0246^ | [2] | 41.47% |  |
|  | BGB = 0.72AGB | [3] |  |  |
| *A. corniculatum* | AGB = 3.1253((CD^2^)H)^0.9063^ | [4] | 41.84% |  |
|  | BGB = 0.403AGB | [3] |  |  |
| *B. gymnorrhiza* | AGB = 0.02804(D^2^H)^1.063^ | [5] | 47.47% |  |
|  | lg(BGB) = 0.912lg(D^2^H)+1.416 | [6] |  |  |

Note: AGB represents aboveground biomass, BGB represents belowground biomass, H refers to plant height, DBH refers to the diameter at breast height measured at 1.3 meters, D refers to basal diameter, and CD refers to crown diameter.

Table S2 Parameters of the UAV and its onboard sensors

| Parameters | Values | Parameters | Values |
| --- | --- | --- | --- |
| Satellite positioning module | GPS + BeiDou + Galileo | Filter | B：450 nm ± 16 nm； |
| Imaging sensor | Six 1/2.9-inch CMOS sensors, effective pixel count of 2.08 million (total pixel count of 2.12 million) |  | G：560 nm ± 16 nm； |
| Resolution | 1600 × 1300 |  | R：650 nm ± 16 nm； |
| Electronic global shutter | 1/100 - 1/20000 s（RGB imaging）；1/100 - 1/10000 s（Multispectral imaging） |  | RE：730 nm ± 16 nm； |
| Focal length | 5.74 mm |  | NIR：840 nm ± 26 nm |

Table S3 The vegetation indices used in this study

| Vegetation Index | Name | Expression | Reference |
| --- | --- | --- | --- |
| NDVI | Normalized Difference Vegetation Index | $\text{NDVI = }\frac{\text{NIR-R}}{\text{NIR+R}}$ | [8] |
| GDNVI | Green Normalized Difference Vegetation Index | $\text{GNDVI = }\frac{\text{NIR-G}}{\text{NIR+G}}$ | [9] |
| DNRE | Normalized Difference Red Edge Vegetation Index | $\text{NDRE = }\frac{\text{NIE-RE}}{\text{NIR+RE}}$ | [10] |
| LCI | Leaf Chlorophyll Index | $\text{LCI = }\frac{\text{NIR-RE}}{\text{NIR+R}}$ | [11] |
| OSAVI | Optimized Soil-Adjusted Vegetation Index | $\text{OSAVI = }\frac{\text{NIR-R}}{\text{NIR+R+0.16}}$ | [12] |
| RVI | Ratio Vegetation Index | $\text{RVI = }\frac{\text{R}}{\text{NIR}}$ | [13] |
| MACI | Modified Anthocyanin Content Index | $\text{MACI = }\frac{\text{NIR}}{\text{G}}$ | [14] |
| VREI | Simple Red Edge Vegetation Index Ratio | $\text{VREI = }\frac{\text{NIR}}{\text{RE}}$ | [15] |
| SR | Simple Ratio | $\text{SR = }\frac{\text{R}}{\text{RE}}$ | - |

Table S4 Adjustment of IDW interpolation parameter

| Serial number | Pixel size | Exponent | Number of points | RMSE（cm） |
| --- | --- | --- | --- | --- |
| IDW1 | 7 | 2 | 12 | 41.32 |
| IDW2 | 3.5 | 2 | 12 | 38.74 |
| IDW3 | 0.5 | 2 | 12 | 43.40 |
| IDW4 | 7 | 3 | 12 | 41.84 |
| IDW5 | 3.5 | 3 | 12 | 38.43 |
| IDW6 | 3.5 | 3 | 6 | 38.15 |
| IDW7 | 3.5 | 3 | 3 | 37.83 |
| IDW8 | 0.1 | 3 | 3 | 37.84 |

Table S5 The expressions of texture feature variables used in this study

| Texture variable | Expression | Reference |
| --- | --- | --- |
| Mean | $\text{Mean = }\sum_{\text{i}\text{,}\text{ j}\text{ }\text{= 0}}^{N \text{- 1}} i\text{P}_{\text{i}\text{,}\text{ j}}$ | [16] |
| Variance | $\text{Var}\text{ = }\sum_{\text{i}\text{,}\text{ j}\text{ }\text{= 0}}^{\text{N }\text{- 1}} \text{iP}_{\text{i,j}}{\text{(}\text{i,j}\text{ -Mean}\text{)}}^{\text{2}}$ |  |
| Entropy | $\text{Ent = }\sum_{\text{i}\text{,}\text{ j}\text{ }\text{= 0}}^{\text{N }\text{- 1}} i\text{P}_{\text{i}\text{,}\text{ j}}\text{(}\text{-ln}\text{P}_{\text{i}\text{,}\text{ j}}\text{)}$ |  |
| Dissimilarity | $\text{Dis = }\sum_{\text{i}\text{,}\text{ j}\text{ }\text{= 0}}^{\text{N }\text{- 1}} \text{i}\text{P}_{\text{i}\text{,}\text{ j}}\left\vert\text{i }\text{- }\text{j} \right\vert$ |  |
| Homogeneity | $\text{Hom =}\sum_{\text{i}\text{,}\text{ j}\text{ }\text{= 0}}^{\text{N }\text{- 1}} i\frac{\text{P}_{\text{i}\text{,}\text{ j}}}{\text{1 + }{\text{(}\text{i}\text{ - }\text{j}\text{)}}^{\text{2}}}$ |  |
| Contrast | $\text{Con = }\sum_{\text{i}\text{,}\text{ j}\text{ = 0}}^{\text{N}\text{ - 1}} i\text{P}_{\text{i}\text{,}\text{ j}}{\text{(}\text{i}\text{ - }\text{j}\text{)}}^{\text{2}}$ |  |
| Correlation | $\text{Cor}\text{ = }\sum_{\text{i}\text{,}\text{ j}\text{ = 0}}^{\text{N}\text{ - 1}} i\text{P}_{\text{i}\text{,}\text{ j}}\left[ \frac{\text{(}\text{i}\text{ }\text{-}\text{Mean}_{\text{i}}\text{) × }\text{(}\text{j}\text{ - }\text{Mean}_{\text{j}}\text{)}}{\sqrt{\text{Variance}_{\text{i}}\text{ }\text{ × }\text{Variance}_{\text{i}}}\text{ }} \right]$ |  |
| Angular Second Moment | $\text{Asm}\text{ = }\sum_{\text{i}\text{,}\text{ j}\text{ = 0}}^{\text{N }\text{- 1}} i{\text{(}\text{P}_{\text{i}\text{,}\text{ j}}\text{)}}^{\text{2}}$ |  |

Note：$\text{P}_{\text{i}\text{，}\text{j}}\text{ = }\frac{\text{V}_{\text{i}\text{，}\text{j}}}{\sum_{\text{i}\text{，}\text{j}\text{ = 0}}^{\text{N }\text{- }\text{1}} \text{V}_{\text{i}\text{，}\text{j}}}$，where $\text{V}_{\text{i}\text{，}\text{j}}$ is the gray level value at the i-th row and j-th column of the moving window, and N is the number of rows or columns.

Table S6 Species identification variables

| Combinations | Variables |
| --- | --- |
| V1 | DN_Red_、DN_Green_、DN_Blue_ |
| V2 | DN_Red_、DN_Green_、DN_Blue_、DN_RE_ |
| V3 | DN_Red_、DN_Green_、DN_Blue_、DN_NIR_ |
| V4 | DN_Red_、DN_Green_、DN_Blue_、DN_RE_、DN_NIR_ |
| V5 | NDVI、GNDVI、OSAVI、RVI、MACI |
| V6 | SR |
| V7 | NDVI、GNDVI、NDRE、LCI、OSAVI、RVI、MACI、VREI、SR |
| V8 | DN_Red_、DN_Green_、DN_Blue_、DSM |
| V9 | SR、DSM |
| V10 | NDVI、GNDVI、OSAVI、RVI、MACI、DSM |
| V11 | NDVI、GNDVI、NDRE、LCI、OSAVI、RVI、MACI、VREI、SR、DSM |
| V12 | DN_Red_、DN_Green_、DN_Blue_、DN_RE_、DN_NIR_、NDVI、GNDVI、NDRE、LCI、OSAVI、RVI、MACI、VREI、SR |
| V13 | DN_Red_、DN_Green_、DN_Blue_、DN_NIR_、NDVI、GNDVI、OSAVI、RVI、MACI、DSM |
| V14 | DN_Red_、DN_Green_、DN_Blue_、DN_RE_、SR、DSM |
| V15 | DN_Red_、DN_Green_、DN_Blue_、DN_RE_、DN_NIR_、NDVI、GNDVI、NDRE、LCI、OSAVI、RVI、MACI、VREI、SR、DSM |

Table S7 Statistics of univariate regression models for carbon stocks of 4 mangrove species

| Species | Expression | Fitting | | Validation | |
| --- | --- | --- | --- | --- | --- |
|  |  | R^2^ | RMSE  （t hm^-2^） | R^2^ | RMSE  （t hm^-2^） |
| *R. stylosa* | AGC = -18.18 + 31.47H_Mean | 0.50 | 35.77 | 0.32 | 42.78 |
|  | BGC = -11.69 + 12.44H_Mean | 0.62 | 11.08 | 0.51 | 12.75 |
| *A. marina* | AGC = -88.61 + 90.95VREI | 0.38 | 6.77 | 0.15 | 8.25 |
|  | BGC = 430.82 -14.59B_Mean | 0.49 | 4.90 | 0.25 | 6.15 |
| *A. corniculatum* | AGC = -464.53 + 20.07RE_Mean | 0.71 | 8.15 | 0.59 | 9.71 |
|  | BGC = -187.21 + 8.09RE_Mean | 0.71 | 3.28 | 0.59 | 3.91 |
| *B. gymnorrhiza* | AGC = 3.31 + 204.83B_Var | 0.84 | 16.35 | 0.44 | 33.04 |
|  | BGC = 5.44 + 59.57B_Var | 0.81 | 5.30 | 0.38 | 10.14 |

Table S8 Statistics of univariate regression models for carbon stocks of mangrove species combinations

| Species | Expression | Fitting | | Validation | |
| --- | --- | --- | --- | --- | --- |
|  |  | R^2^ | RMSE  （t hm^-2^） | R^2^ | RMSE  （t hm^-2^） |
| *R. stylosa*-*A. corniculatum* | AGC = -7.43 + 28.74H_Mean | 0.67 | 26.78 | 0.59 | 30.01 |
|  | BGC = -1.46 + 10.00H_Mean | 0.69 | 8.82 | 0.63 | 9.76 |
| *R. stylosa*-*A. marina* | AGC = -28.49 + 33.50H_Mean | 0.69 | 26.55 | 0.62 | 29.80 |
|  | BGC = 2.75 + 9.20H_Mean | 0.57 | 9.51 | 0.48 | 10.48 |
| *R. stylosa*-*B. gymnorrhiza* | AGC = 18.23 + 22.19H_Mean | 0.28 | 40.69 | 0.15 | 44.65 |
|  | BGC = 4.62 + 8.41H_Mean | 0.36 | 12.72 | 0.25 | 13.89 |
| *A. marina*-*A. corniculatum* | AGC = 370.66 -11.95B_Mean | 0.30 | 10.68 | 0.17 | 11.83 |
|  | BGC = -0.18 + 11.15H_Mean | 0.46 | 5.44 | 0.35 | 5.96 |
| *A. marina*-*B. gymnorrhiza* | AGC = -7.17 + 198.90B_Var | 0.65 | 20.39 | 0.04 | 35.21 |
|  | BGC = 8.85 + 51.23B_Var | 0.53 | 6.78 | 0.02 | 10.10 |
| *A. corniculatum*-*B. gymnorrhiza* | AGC = -369.21 + 16.07RE_Mean | 0.56 | 22.72 | 0.23 | 30.84 |
|  | BGC = -108.65 + 4.89RE_Mean | 0.55 | 7.06 | 0.27 | 9.18 |
| *R. stylosa*-*A. marina*-*A. corniculatum* | AGC = -15.36 + 29.99H_Mean | 0.68 | 23.53 | 0.61 | 25.94 |
|  | BGC = 1.64 + 9.45H_Mean | 0.63 | 8.24 | 0.57 | 8.91 |
| *R. stylosa*-*A. marina*-*B. gymnorrhiza* | AGC = -3.90 + 27.17H_Mean | 0.39 | 35.62 | 0.31 | 38.10 |
|  | BGC = 6.21 + 8.09H_Mean | 0.38 | 10.93 | 0.30 | 11.66 |
| *R. stylosa*-*A. corniculatum*-*B. gymnorrhiza* | AGC = 6.15 + 25.15H_Mean | 0.41 | 34.35 | 0.33 | 36.68 |
|  | BGC = 2.79 + 8.86H_Mean | 0.47 | 10.75 | 0.40 | 11.44 |
| *A. marina*-*A. corniculatum*-*B. gymnorrhiza* | AGC = -290.43 + 12.97RE_Mean | 0.43 | 22.66 | 0.16 | 28.29 |
|  | BGC = 88.42 - 525.93RE_Asm | 0.26 | 8.20 | 0.15 | 8.91 |
| *R. stylosa*-*A. marina*-*A. corniculatum*-*B. gymnorrhiza* | AGC = -3.17 + 26.84H_Mean | 0.44 | 31.41 | 0.38 | 33.18 |
|  | BGC = 4.13 + 8.64H_Mean | 0.45 | 9.82 | 0.40 | 10.34 |

Table S9 Statistics of multivariate regression models for AGC of mangrove species combinations

| Species | Expression | Fitting | | Validation | |
| --- | --- | --- | --- | --- | --- |
|  |  | R^2^ | RMSE  （t hm^-2^） | R^2^ | RMSE  （t hm^-2^） |
| *B. gymnorrhiza* | AGC = 17.14 + 293.07B_Var - 31.21 R_Con | 0.95 | 9.57 | 0.91 | 12.61 |
| *R. stylosa*-*B. gymnorrhiza* | AGC = -1208.45 + 25.88H_Mean + 49.80G_Mean + 96.49B_Cor | 0.66 | 28.09 | 0.43 | 36.45 |
| *A. marina*-*A. corniculatum* | AGC = 844.33 - 25.88B_Mean + 61.54NIR_Cor - 116.35B_Hom | 0.77 | 6.08 | 0.64 | 7.78 |
| *A. corniculatum*-*B. gymnorrhiza* | AGC = -605.25 + 26.63RE_Mean -2.64NIR_Con | 0.66 | 19.80 | 0.31 | 29.06 |
| *R. stylosa*-*A. marina*-*B. gymnorrhiza* | AGC = -39.60 + 33.46H_Mean + 98.44R_Var -77.21B_Con  + 110.40R_Cor | 0.70 | 0.65 | 0.50 | 32.62 |
| *R. stylosa*-*A. corniculatum*-*B. gymnorrhiza* | AGC = -727.02 + 37.09H_Mean + 29.26G_Mean | 0.54 | 0.51 | 0.36 | 36.48 |
| *B. gymnorrhiza*-*A. marina*-*A. corniculatum* | AGC = -269.84 + 11.82RE_Mean + 75.27NIR_Cor | 0.51 | 0.47 | 0.22 | 27.24 |
| *R. stylosa*-*B. gymnorrhiza*-*A. marina*-*A. corniculatum* | AGC = -207.11 + 23.60H_Mean + 9.40RE_Mean + 72.15B_Cor  - 146.90R_Asm | 0.67 | 0.63 | 0.50 | 29.94 |

Table S10 Statistics of multivariate regression models for BGC of mangrove species combinations

| Species | Expression | Fitting | | Validation | |
| --- | --- | --- | --- | --- | --- |
|  |  | R^2^ | RMSE  （t hm^-2^） | R^2^ | RMSE  （t hm^-2^） |
| *B. gymnorrhiza* | BGC = -54.10 + 95.55B_Var + 241.07NIR_Hom -20.28B_Con | 0.93 | 3.23 | 0.95 | 3.94 |
| *A. marina* | BGC = 296.90 - 10.55B_Mean + 48.76NIR_Cor + 9.03H_Mean | 0.88 | 2.37 | 0.62 | 4.46 |
| *R. stylosa*-*B. gymnorrhiza* | BGC = -236.37 + 7.69H_Mean + 13.04G_Mean + 47.68B_Cor -241.76RVI -105.47G_Hom | 0.82 | 6.85 | 0.69 | 4.23 |
| *A. marina*-*A. corniculatum* | BGC = -1.55 + 11.82H_Mean + 39.77RE_Cor -13.23B_Cor | 0.79 | 3.38 | 0.69 | 4.23 |
| *A. corniculatum*-*B. gymnorrhiza* | BGC = -180.48 + 8.22RE_Mean -2.38NIR_Con + 2.70RE_Var | 0.87 | 3.81 | 0.42 | 11.42 |
| *A. marina*-*B. gymnorrhiza* | BGC = 11.32 + 80.25B_Var - 8.92R_Con | 0.68 | 5.61 | 0.53 | 6.96 |
| *R. stylosa*-*A. corniculatum*-*B. gymnorrhiza* | BGC = -117.71 + 9.18H_Mean + 6.02RE_Mean + 25.92B_Cor - 81.53R_Asm - 1.02NIR_Con | 0.80 | 6.56 | 0.67 | 8.56 |
| *R. stylosa*-*B. gymnorrhiza*-*A. marina*-*A. corniculatum* | BGC = 12.89 + 10.17H_Mean - 38.70R_Asm | 0.51 | 9.28 | 0.44 | 10.00 |

Table S11 Vegetation carbon stock of mangrove plants in Yingluo Bay, Guangxi

| Species | Carbon stock（t hm^-2^） | Supplement | Reference |
| --- | --- | --- | --- |
| *A. corniculatum*-*A. marina* | 50.98 ± 8.82 | 15-year-old | [17] |
| *A. corniculatum*-*A. marina*-*R. stylosa* | 289.9 ± 18.33 | 80-year-old |  |
| *A. corniculatum* | 45.25 | High, medium, and low tidal level sampling points | [18] |
| *A. marina* | 37.78 | High, medium, and low tidal level sampling points |  |
| *B. gymnorrhiza* | 61.95 | High, medium, and low tidal level sampling points |  |
| *R. stylosa* | 122.61 | High, medium, and low tidal level sampling points |  |
| *A. corniculatum* | 24.41 | Medium tidal level | [18] |
| *A. marina* | 33.87 | Medium tidal level |  |
| *B. gymnorrhiza* | 84.92 | High tidal level |  |
| *R. stylosa* | 137.52 | High tidal level |  |
| *A. corniculatum* | 55.66 ± 20.57 | High, medium, and low tidal level sampling zones | [19] |
| *A. corniculatum*-*A. marina*-*R. stylosa* | 51.46 ± 34.52 | High, medium, and low tidal level sampling zones |  |
| *A. marina* | 42.07 ± 13.86 | High, medium, and low tidal level sampling zones |  |
| *R. stylosa* | 167.33 ± 36.77 | High tidal level | [20] |
| *B. gymnorrhiza* | 97.76 ± 39.62 | High tidal level |  |
| *A. marina* | 24.81 ± 12.44 | Low tidal level |  |


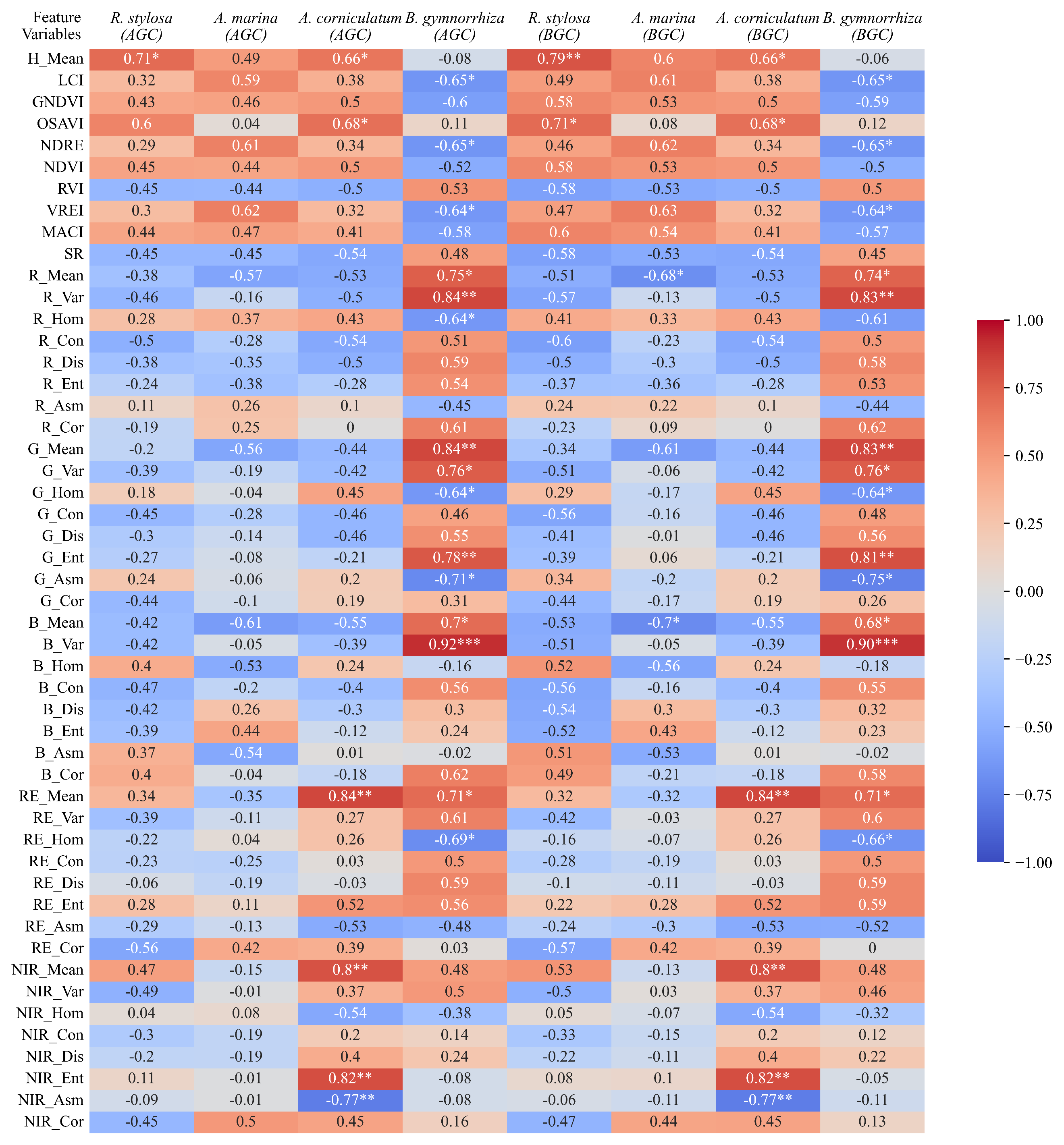


Fig. S1 Pearson correlation (Pearson’s r) between above-ground carbon stocks/below-ground carbon stocks and feature variables of mangrove plants. *** indicates a significant correlation at the 0.001 level (two-tailed); ** indicates a significant correlation at the 0.01 level (two-tailed); * indicates a significant correlation at the 0.05 level (two-tailed).


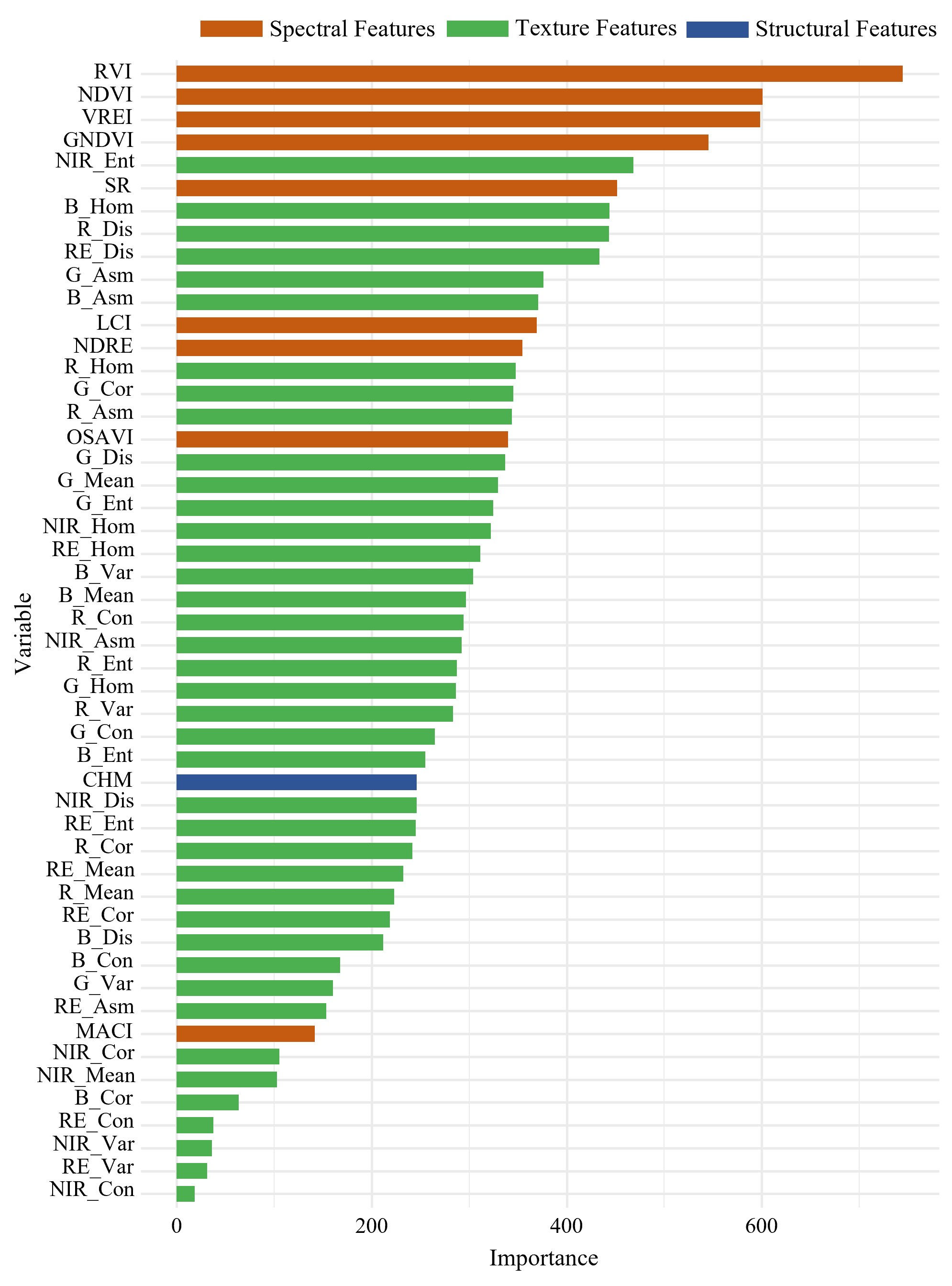


Fig. S2 The importance of spectral, textural and structural feature variables in mangrove species identification.

**Reference**

1. Comley, B. W. T., & McGuinness, K. A. Above-and below-ground biomass, and allometry, of four common northern Australian mangroves. *Australian Journal of Botany*, **53(5)**, 431-436 (2005). https://doi.org/10.1071/BT04162
2. Fan H, Yin Y, & Lao L. Correlation analysis of aboveground biomass of Avicennia marina mangrove plants on the Guangxi coast. *Journal of the Guangxi Academy of Sciences*, **9(2)**, 25-30 (1993).
3. Tam N. F. Y., Wong Y. S., Lan C. Y., & Chen G. Z. Community structure and standing crop biomass of a mangrove forest in Futian Nature Reserve, Shenzhen, China. *Hydrobiologia*, **295** 193-201 (1995). https://doi.org/10.1007/BF00029126
4. Fu, W., & Wu, Y. Estimation of aboveground biomass of different mangrove trees based on canopy diameter and tree height. *Procedia Environmental Sciences*, **10**, 2189-2194 (2011). https://doi.org/10.1016/j.proenv.2011.09.343
5. Deshar, R., Sharma, S., Hoque, A. T. M. R., Mouctara, K., & Hagihara, A. Self-thinning of leaf, wood and aboveground in overcrowded mangrove *Bruguiera gymnorrhiza* stands in Okinawa Island, Japan. *Procedia Environmental Sciences*, **13**, 982-993 (2012). https://doi.org/10.1016/j.proenv.2012.01.091
6. Zhu K., Liao B., & Zhang J. Studies on the biomass of mangrove plantation of *Sonneratia apetala* and *Bruguiera gymnorrhiza* in the wetland of Nansha in Guangzhou City. *Forest Research*, **24(4)**, 531-536 (2011).
7. Cao, Q. The estimation of mangrove biomass and carbon storage using remote sensing data in Beibu Gulf Coast. *Chinese Academy of Forestry* (2010).
8. Rouse J., Haas R. H., Schell J. A., & Deering D W. Monitoring vegetation systems in the Great Plains with ERTS. *Washington: Scientific and Technical Information Office, National Aeronautics and Space Administration*, **1**, 309-317 (1974).
9. Gitelson, A. A., Kaufman, Y. J., & Merzlyak, M. N. Use of a green channel in remote sensing of global vegetation from EOS-MODIS. *Remote sensing of Environment*, **58(3)**, 289-298 (1996). https://doi.org/10.1016/S0034-4257(96)00072-7
10. Gitelson, A., & Merzlyak, M. N. Quantitative estimation of chlorophyll-a using reflectance spectra: Experiments with autumn chestnut and maple leaves. *Journal of Photochemistry and Photobiology B: Biology*, **22(3)**, 247-252 (1994). https://doi.org/10.1016/1011-1344(93)06963-4
11. Zebarth B. J., Younie M., Paul J. W., & Bittman S. Evaluation of leaf chlorophyll index for making fertilizer nitrogen recommendations for silage corn in a high fertility environment. *Communications in Soil Science and Plant Analysis*, **33** 665-684 (2002). https://doi.org/10.1081/CSS-120003058
12. Rondeaux G., Steven M., & Baret F. Optimization of soil-adjusted vegetation indices. *Remote Sensing of Environment*, **55**, 95-107 (1996). https://doi.org/10.1016/0034-4257(95)00186-7
13. Jordan, C. F. Derivation of leaf‐area index from quality of light on the forest floor. *Ecology*, **50(4)**, 663-666 (1969). https://doi.org/10.2307/1936256
14. Shendryk Y., et al. Fine-scale prediction of biomass and leaf nitrogen content in sugarcane using UAV LiDAR and multispectral imaging. *International Journal of Applied Earth Observation and Geoinformation*, **92** 102177 (2020). https://doi.org/10.1016/j.jag.2020.102177
15. Vogelmann J. E., Rock B. N., & Moss D. M. Red edge spectral measurements from sugar maple leaves. *International Journal of Remote Sensing*, **14** 1563-1575 (1993). https://doi.org/10.1080/01431169308953986
16. Haralick R. M., Shanmugam K., & Dinstein I. Textural features for image classification. *IEEE Transactions on systems, man, and cybernetics*, **6**, 610-621 (1973). https://doi.org/10.1109/TSMC.1973.4309314
17. Yu C., et al. Development of ecosystem carbon stock with the progression of a natural mangrove forest in Yingluo Bay, China. *Plant and Soil* **460**, 391-401 (2021). <https://doi.org/10.1007/s11104-020-04819-3>
18. Wang G., Guan D., Xiao L., & Peart M. R. Ecosystem carbon storage affected by intertidal locations and climatic factors in three estuarine mangrove forests of South China. *Regional Environmental Change*, **19(6)**, 1701-1712 (2019). https://doi.org/10.1007/s10113-019-01515-6
19. Wang G., et al. Spatial patterns of biomass and soil attributes in an estuarine mangrove forest (Yingluo Bay, South China). *European Journal of Forest Research* **133**, 993-1005 (2014). <https://doi.org/10.1007/s10342-014-0817-3>
20. Wang G., Guan D., Peart M. R., Chen Y., & Peng Y. Ecosystem carbon stocks of mangrove forest in Yingluo Bay, Guangdong Province of South China. *Forest Ecology and Management*, **310**, 539-546 (2013). https://doi.org/10.1016/j.foreco.2013.08.045
